# Comparing Cyclicity Analysis With Pre-established Functional Connectivity Methods to Identify Individuals and Subject Groups Using Resting State fMRI

**DOI:** 10.3389/fncom.2019.00094

**Published:** 2020-01-20

**Authors:** Somayeh Shahsavarani, Ivan T. Abraham, Benjamin J. Zimmerman, Yuliy M. Baryshnikov, Fatima T. Husain

**Affiliations:** ^1^Beckman Institute for Advanced Science and Technology, University of Illinois at Urbana-Champaign, Champaign, IL, United States; ^2^Department of Speech and Hearing Science, University of Illinois at Urbana-Champaign, Champaign, IL, United States; ^3^Neuroscience Program, University of Illinois at Urbana-Champaign, Champaign, IL, United States; ^4^Department of Electrical & Computer Engineering, University of Illinois at Urbana-Champaign, Champaign, IL, United States; ^5^Department of Mathematics, University of Illinois at Urbana-Champaign, Champaign, IL, United States

**Keywords:** resting state fMRI, cyclicity analysis, functional interactivity, tinnitus, classification, fingerprinting, latent space, data augmentation

## Abstract

The resting state fMRI time series appears to have cyclic patterns, which indicates presence of cyclic interactions between different brain regions. Such interactions are not easily captured by pre-established resting state functional connectivity methods including zero-lag correlation, lagged correlation, and dynamic time warping distance. These methods formulate the functional interaction between different brain regions as similar temporal patterns within the time series. To use information related to temporal ordering, cyclicity analysis has been introduced to capture pairwise interactions between multiple time series. In this study, we compared the efficacy of cyclicity analysis with aforementioned similarity-based techniques in representing individual-level and group-level information. Additionally, we investigated how filtering and global signal regression interacted with these techniques. We obtained and analyzed fMRI data from patients with tinnitus and neurotypical controls at two different days, a week apart. For both patient and control groups, we found that the features generated by cyclicity and correlation (zero-lag and lagged) analyses were more reliable than the features generated by dynamic time warping distance in identifying individuals across visits. The reliability of all features, except those generated by dynamic time warping, improved as the global signal was regressed. Nevertheless, removing fluctuations >0.1 Hz deteriorated the reliability of all features. These observations underscore the importance of choosing appropriate preprocessing steps while evaluating different analytical methods in describing resting state functional interactivity. Further, using different machine learning techniques including support vector machines, discriminant analyses, and convolutional neural networks, our results revealed that the manifestation of the group-level information within all features was not sufficient enough to dissociate tinnitus patients from controls with high sensitivity and specificity. This necessitates further investigation regarding the representation of group-level information within different features to better identify tinnitus-related alternation in the functional organization of the brain. Our study adds to the growing body of research on developing diagnostic tools to identify neurological disorders, such as tinnitus, using resting state fMRI data.

## 1. Introduction

In this paper, we further evaluate the efficacy of cyclicity analysis (Baryshnikov and Schlafly, 2016) that we have previously used to characterize the interaction between brain regions (Zimmerman et al., 2018). We compare this novel technique with pre-established functional connectivity techniques including zero-lag correlation, lagged correlation, and dynamic time warping distance using resting state functional magnetic resonance imaging (fMRI) data.

The human brain generates spontaneous neuronal activity even at rest–in the absence of any stimulus, motor behavior, or cognitive task. Such brain activity can be measured by blood oxygenation level dependent (BOLD) signals using fMRI. Previous studies have showed that these spontaneous activities are not just random, but that they form spatial patterns of coherent networks, indicating an interaction between neuronal ensembles. (Biswal et al., 1995; Xiong et al., 1999; Greicius et al., 2003; Horwitz, 2003; Fox et al., 2005; Fransson, 2005; Vincent et al., 2006).

Resting state functional connectivity (rsFC) is one of the most widely used analysis methods to specify intrinsic neural interactivity. Functional connectivity is defined as simultaneous or similar activity in discrete brain regions, which are not necessarily adjacent to one another (Rubinov and Sporns, 2010). The most popular rsFC technique measures temporal correlation of the BOLD time series of the regions of interest (ROIs), assuming static zero-lag synchrony (Hampson et al., 2002). Despite being a core subroutine of rsFC, the correlation analysis has two main drawbacks. First, it assumes the time series are not auto-correlated (Dean and Dunsmuir, 2016), but this assumption may not hold for resting state BOLD signals (Arbabshirani et al., 2014). Second, the correlation analysis fails to capture the information expressed in lag structure (Jafri et al., 2008; Mitra et al., 2014). Lagged correlation (Mitra et al., 2014) and dynamic time warping (DTW) distance (Sakoe and Chiba, 1978; Meszlényi et al., 2017b) are two alternative techniques to address the latter drawback. Although these techniques take the phase shift between the time series into account, they have yet to fully benefit from the information manifested within lag structure. One such piece of information (manifested in lag structure but not utilized by these techniques) is collective temporal ordering of time series. The resting state fMRI time series demonstrate cyclic patterns, which indicates an underlying self-sustained cycling among processes in different ROIs. As explained by Baryshnikov and Schlafly (2016), there is a distinction between cyclic and periodic phenomena. Similar to periodic signals, cyclic signals demonstrate repetitive patterns, albeit not in a periodic fashion. That is, the cyclic patterns are not invariant for any time interval, e.g., *T*. It is worth mentioning that periodic phenomena are necessarily cyclic, but cyclic phenomena are not periodic. For more details, see Baryshnikov and Schlafly (2016).

The cyclic relationships between brain regions remain indiscernible to correlation-based and DTW distance measures. Based on the notion of cyclic signals (Baryshnikov and Schlafly, 2016), cyclicity analysis technique has been used (Zimmerman et al., 2018) to assess resting state functional connectivity by examining the temporal ordering, i.e., leader-follower relationship, between neural activity of different ROIs, providing information germane to the cyclic ordering among the time series. Unlike effective connectivity techniques, such as Granger causality (Hamilton et al., 2011; Yu et al., 2016), cyclicity analysis does not reveal causal influence between brain regions although it provides us with information flow. Granger causality analysis is a linear multivariate autoregressive model that assumes resting state fMRI time series are stationary, which may not be an accurate assumption (Chang and Glover, 2010). In contrast, cyclicity analysis is a non-linear technique, invariant to re-parameterization and does not require the stationary assumption. In correlation-based and DTW distance measures, the correlation and distance values determine the extent of neural connectivity. In cyclicity analysis, however, the sign and magnitude of the values indicate an average directed leader-follower relationship and its reverse; while a zero value implies zero average interaction. We group all these techniques together in a term broader than functional connectivity: “resting state functional interactivity” (rsFI), encompassing all manner of direct and indirect interactions among brain regions.

The lag differences between resting state time series may carry information pertinent to intrinsic neuronal fluctuations (Mitra and Raichle, 2016). As alluded earlier, lagged correlation analysis has been employed to mitigate the impact of phase shifts on rsFI (Jafri et al., 2008; Mitra et al., 2015). Although lagged correlation provides information useful to understanding the temporal organization of resting state BOLD signals, the general low temporal resolution of fMRI still poses practical hindrances to this technique. That is, the time lag between time series may be on the order of less than the temporal sampling, necessitating the use of interpolation methods. This leaves open the exact interpolation method used as a hyper-parameter to be chosen, which introduces some degree of uncertainty. In Mitra et al. (2014, 2015), the authors used lagged cross-correlation to study spatiotemporal resting state BOLD fluctuations assuming that cross-correlation curves exhibit only a single peak at a certain time delay. However, this assumption depends on the preprocessing steps applied to the time series.

Unlike correlation, the DTW distance takes into account non-stationary phase lags between ROIs, capturing functional interactivity with greater sensitivity. The DTW distance measures the similarity between two time series by finding their optimal alignment under non-linear but monotonic warping of the time axis (Ding et al., 2008). Over the past decade, this measure has attracted attention in neuroscientific applications. Silbert et al. (2014) used DTW to align fMRI time series with speech signals and in a multi-modal study, Dinov et al. (2016) applied DTW on an EEG-fMRI data to study brain dynamics, assessing oscillatory patterns. Recently, Meszlényi et al. (2017b) used DTW to measure rsFC and classify subject groups based on gender while examining different machine learning techniques including support vector machines and convolutional neural networks. Their results showed that DTW was more robust and had higher sensitivity to group differences compared with zero-lag correlation and lagged correlation. Despite this noted advantage of the DTW distance measure over correlation-based analyses, its efficacy in rsFI analysis has yet to be well-explored.

Unlike zero-lag and lagged correlation analyses (both of which aim to find similarity between signals) and DTW distance measures (which aim to find similarities between signals while accounting for non-linear warping), the cyclicity analysis determines pairwise influences between time series. Cyclicity analysis (Baryshnikov and Schlafly, 2016) is a new technique for extracting features invariant to time re-parameterizations and translations of signals. In Baryshnikov and Schlafly (2016), the authors introduced simple modifications to the analysis accounting for noisy data and discussed its applicability to signals with aperiodic but repetitive patterns, which are properties abundant in physiological data. Cyclicity analysis uses so-called iterated path integrals, which are also systematically exploited in the “signature method” (Chevyrev and Kormilitzin, 2016). Whereas the general idea of replacing the time series by their signatures faces the challenge of exponential increase in the dimensionality of features, moving from all pairs, to all triplets, to quadruplets, and so on, cyclicity analysis restricts itself to pairwise influences so that the generated features are one, amenable to matrix analysis methods, and two, maintain a balance between the number of independent samples and dimensionality of features. This allows it to remain interpretable and suitable to machine learning techniques when there is a dearth of data as in the case of fMRI studies. In our previous study (Zimmerman et al., 2018), we applied the cyclicity analysis to resting state fMRI data and showed that the features extracted from this technique are relatively stable apropos of time and subjects.

To use rsFI measures as an objective biomarker aiming to develop diagnostic tools, it is necessary to prove the stability of such markers across time. Using zero-lag correlation analysis and data from the Human Connectome Project, Finn et al. (2015) and Amico and Goñi (2018) showed the stability and reliability of the fMRI functional interactivity profile as a “fingerprint” to identify individuals from a healthy population, for both resting state and task-based paradigms. Using cyclicity analysis, Zimmerman et al. (2018) also showed that individuals from both patient and healthy control populations can be reliably identified based on the resting state fMRI data collected across a week's span. To the best of our knowledge, similar evaluation has not yet been conducted to examine the stability of lagged correlation and DTW distance as compared with cyclicity and zero-lag correlation analyses. Additionally, assessing the effect of different preprocessing steps, especially the effect of the controversial ones, on rsFI measures is of great importance as it provides insights about both the information embedded in each rsFI measure and various preprocessing steps. One such preprocessing step is global signal regression (GSR) (Murphy and Fox, 2017). Global signal is referred to as spontaneous BOLD fluctuations that are common throughout the brain and defined as the average of BOLD signals over all voxels in the brain (Fox et al., 2009; Murphy et al., 2009). The fluctuations in the global signal have been attributed to non-neuronal origins, especially physiologically induced fluctuations, such as changes in the level of arterial carbon dioxide or changes in cardiac rhythm. In the literature, it has been argued that the variances associated with global signal should be removed from the resting state data because it may inflate the connectivity measures, specifically the results from the correlation metric (Fox et al., 2009). Murphy et al. (2009), however, showed that regressing out the fluctuations related to global signal introduces spurious negative correlations as well as reducing correlation in some areas. In contrast, Fox et al. (2009) argue that using GSR indeed enhances and improves results from correlation analysis. This lack of consensus across studies necessitates the assessment of functional interactivity techniques with and without GSR by each study (Caballero-Gaudes and Reynolds, 2017). In a recent study, for instance, Meszlényi et al. (2017b) evaluated the efficacy of DTW distance as a rsFC measure by comparing it with correlation (both zero-lagged and lagged) while considering both conditions of with and without GSR. Their results demonstrated that the DTW distance is more robust to employing GSR than the correlation analyses.

Neurological disorders, such as Alzheimer's disease (Greicius et al., 2004), depression (Greicius et al., 2007), and schizophrenia (Zhou et al., 2007) disturb resting state coherence patterns and reorganize the interaction between different parts of the brain. Likewise, such disturbances have been reported in patients with tinnitus (Schmidt et al., 2017). Tinnitus or “ringing in the ears” refers to phantom auditory perception when there is no external physical source; a condition affecting more than 50 million adults in the United States (Shargorodsky et al., 2010). The handicap resulting from tinnitus varies from mild to severe. Depending on the level of severity, tinnitus can become bothersome, leading to distress, frustration, annoyance, disrupted sleep, depression, and/or anxiety, having a major impact on the patients' personal and professional life (Møller, 2007). Tinnitus is often subjectively diagnosed by self-report. That is, there are no standard objective criteria to corroborate the patients' anecdotal report of tinnitus presence, and there is no cure for this condition. Previous studies have shown that pathological disturbances in the coherence of spontaneous BOLD fluctuations hold valuable diagnostic information to discriminate between subject groups. Our previous study (Zimmerman et al., 2018), using cyclicity analysis, showed promising results in finding group level differences between patients with tinnitus and healthy controls, suggesting value in using resting state BOLD signals as a diagnostic tool to objectively dissociate tinnitus from the norm.

### 1.1. Goal of Study

The goal of the current study was to compare cyclicity analysis with pre-established rsFI techniques including zero-lag correlation, lagged correlation, and DTW distance. To demonstrate the practical and clinical relevance of this comparison, differences between tinnitus patients and controls were assessed with respect to rsFI. Further, we examined the stability of the features extracted by these four measures across two fMRI scans, conducted 1 week apart. We evaluated the effect of preprocessing steps on these measures; specifically, we investigated the effect of GSR and filtering on resting state fMRI data. We compared the classification accuracy using these features under traditional and modern machine learning techniques including support vector machines, discriminant analysis, and convolutional neural networks.

## 2. Functional Interactivity Measures

In this section, we briefly introduce the technical definition of cyclicity analysis, lagged correlation analysis, and dynamic time warping distance measure.

### 2.1. Cyclicity Analysis

Tools from Fourier analysis can be employed to investigate the properties of periodic signals. Precisely, a signal, *r*(*t*), is considered periodic if its patterns repeat regularly. In other words, there is a time interval *T* such that *r*(*t* + *T*) = *r*(*t*). However, many physiological signals, such as cardiac rhythm, electroencephalogram recordings, and fMRI BOLD signals are aperiodic, which are not truly periodic yet have repetitive patterns across time. Therefore, Fourier analysis cannot be applied to investigate the properties of cyclic signals where the patterns repeat irregularly. Instead, cyclicity analysis assumes the existence of an appropriate re-parameterization of time under which unobserved periodic signals generate the observed aperiodic ones. It does this by interpreting the observed time series as paths in path spaces. Consequently, they can be analyzed using re-parameterization invariant features of paths and path spaces, where the cyclic signals are considered as paths. In fact, a signal, *g*(*t*), is considered cyclic if there is a monotonically increasing bounded function, ϕ(*t*), so that *g*(ϕ(*t*)) is periodic. For more details, see our online tutorial: http://acnlab.beckman.illinois.edu/.

Cyclicity analysis aims to infer the temporal ordering structure in such cases by analyzing all pairs of signals in the *N* dimensional multivariate time series. For given two scalar valued signals *f*(*t*) and *g*(*t*) observed over an interval [0, *T*], equivalued at 0 and *T*, one can assign a signed algebraic area as

(1) $$\begin{array}{r} A_{fg}:=\frac{1}{2}\overset{T}{\underset{0}{\int }}g\left(t\right)df\left(t\right)-f\left(t\right)dg\left(t\right)dt, \end{array}$$

to the pair over the interval of observation. The magnitude of this quantity is determined by the area enclosed by projection of the trajectory (*f*(*t*), *g*(*t*))^*T*^ onto the *f*−*g* coordinate planes with the sign positive if *g*(*t*) follows *f*(*t*).

For any given *N* dimensional times series, this leads to the creation of a skew-symmetric lead matrix *A* with $\frac{N\left(N-1\right)}{2}$ independent entries. Each element *A*_*kl*_ of such a matrix corresponds to the average leader follower relationship (implied by the sign and magnitude of the calculated area) between two pairs or ROI time series: *X*_*k*_ and *X*_*l*_ over the interval of observation. The spectral analysis of the lead matrix provides information pertinent to collective temporal ordering of the signals even in the presence of noise. That is, the temporal ordering is associated with the phase angles of the complex valued elements in the eigenvector corresponding to the largest eigenvalue. For more details, see Baryshnikov and Schlafly (2016); Zimmerman et al. (2018).

### 2.2. Lagged Correlation Analysis

Zero-lag correlation analysis assumes synchronous activity between different ROIs and fails to extract information from the latency structure (Majeed et al., 2011; Mitra et al., 2014). Lagged correlation analysis has been suggested to take latency into account while measuring the correlation between time series. Lagged correlation analysis leads to two matrices from the underlying time-series data: (1) lagged correlation matrix (LCM), and (2) time delay matrix (TDM). For every pair of time series, *x*(*t*) and *y*(*t*), the correlation between *x*(*t* ± τ) and *y*(*t*) is computed. The value of τ at which the correlation function has extremum is stored in the TDM and the corresponding correlation value is stored in the LCM.

### 2.3. Dynamic Time Warping

The dynamic time warping (DTW) algorithm quantifies the distance between two time series, namely their overall similarities in shape (e.g., peaks and troughs) by taking temporal dynamics, such as speed or phase shift into account. The DTW algorithm was first introduced by Sakoe and Chiba (1978) in the realm of automatic speech recognition. The key idea is that some non-linear variations of speech signals, such as temporal compression, local delays, etc., can and sometimes must be ignored. For example, the frequency and the rate at which a sentence is uttered is immaterial and needs to be ignored while the sentence is being semantically decoded. The DTW algorithm computes the distance between two given time series of not necessarily equal length, *X* = (*x*_1_, *x*_2_, …, *x*_*k*_, …, *x*_*M*_) and *Y* = (*y*_1_, *y*_2_, …, *y*_*j*_, …, *y*_*N*_), by finding the optimal alignment between the series. In doing so, it constructs an *M* by *N* matrix, *C*. Each element of the matrix *C*_*kj*_ corresponds to the optimal cost of the alignment between *X*_*k*_ = (*x*_1_, *x*_2_, …, *x*_*k*_) and *Y*_*j*_ = (*y*_1_, *y*_2_, …, *y*_*j*_), i.e., the first *k* points of *X* and the first *j* points of *Y*. This optimal cost is obtained as

(2) $$\begin{array}{r} C_{kj}=d_{kj}+\min\left(C_{\left(k-1\right)j},C_{\left(k-1\right)\left(j-1\right)},C_{k\left(j-1\right)}\right), \end{array}$$

where *d*_*kj*_ is a distance metric, such as the Euclidean distance. One, of course, has a choice of a distance function. The DTW algorithm, in fact, calculates the “optimal global match” between the index sets {*k*} and {*j*} while satisfying the constraints, such as monotonicity and continuity. See Meszlényi et al. (2016, 2017b) for more details. To avoid the brute-force search of all possible alignments for the optimal match, dynamic programming-based approach has been used to implement the DTW with asymptotic running time of *O*(*MN*). The optimal alignment is defined as a warping path, which is the sequence of indices $\left\{\tilde{k}\right\}$ on *X* and $\left\{\tilde{j}\right\}$ on *Y* such that $C_{\tilde{k}\tilde{j}}$ is minimal for each corresponding pair. The warping path of two identical series is defined by the diagonal indices. As the distance between the two series increases, the warping path deviates from the diagonal. High computational complexity is the main hurdle that limits the applicability of this technique.

## 3. Materials and Methods

### 3.1. Participants and Data Acquisition

Two groups of participants were included in this study: those with tinnitus as a patient group and those without tinnitus as a control group. The tinnitus group included 50 patients (mean age ± standard deviation: 52.96 ± 10.29 years; 38 with hearing loss; 21 women; mean tinnitus duration: 15.5 ± 14.02 years) and the control group 29 participants (mean age: 47.75 ± 11.06 years; 12 with hearing loss; 15 women). Resting state BOLD data were obtained using a 3T Siemens Magnetom Prisma MRI scanner in two visits, 1 week apart. To do so, a gradient echo-planar EPI sequence with transversal orientation was used (repetition time [TR] = 2000 ms, echo time [TE] = 25 ms, flip angle = 90°, 38 slices, voxel size = 2.5 × 2.5 × 3 mm^3^). In addition to functional images, structural images including a high-resolution, T1-weighted sagittal MPRAGE image (TR = 2,300 ms, TE = 2.32 ms, flip angle = 8°, 192 slices, voxel size = 0.9 × 0.9 × 0.9 mm^3^), and a lower-resolution, T2-weighted transversal image (TR = 3,400 ms, TE = 65 ms, flip angle = 120°, 38 slices, voxel size = 1.2 × 1.2 × 3 mm^3^), were collected and further used in preprocessing. At each visit, two ~10-min resting state data were acquired while participants were lying supine inside the scanner with eyes open looking at a white fixed point (+) on the center of a black screen. Further, earplugs were used to reduce the scanner noise. Data collection was conducted with the approval of the University of Illinois at Urbana-Champaign Institutional Review Board (#15955) and each participant provided informed consent prior to image acquisition in the first visit.

### 3.2. Preprocessing

Data were preprocessed using Statistical Parametric Mapping software (SPM12) (http://www.fil.ion.ucl.ac.uk/spm/software/spm12). The first four volumes of resting state data were excluded to allow for magnet stabilization, leaving 300 resting state volumes for preprocessing at each run.

Similar to our previous work (Schmidt et al., 2017; Zimmerman et al., 2018), data were first corrected for slice timing, and then realigned to the mean fMRI image to correct for head motion using a 6-parameter rigid body transformation. The data with motion greater than a 2-mm translation or 2° rotation were removed from resting state analysis. Subsequently, two co-registration steps were carried out using a 12-parameter affine transformation: (1) aligning the T-2 weighted image to the mean fMRI image, and (2) aligning the T-1 weighted image to the co-registered T-2 weighted image. Next, the co-registered T-1 weighted was normalized to a stereotactic space, i.e., MNI space, using a non-linear warp transformation. This image was used to realign and normalize the functional data. Further, the normalized images were spatially smoothed using a Gaussian kernel (8×8×8 mm^3^). The segmentation step was not performed. MarsBaR (Brett et al., 2002), a toolbox for SPM12, was used to extract the time series with 300 time points from ROIs. The 33 ROIs were chosen based on our previous studies of tinnitus (Zimmerman et al., 2018), and are given in Table 1. The time series of each ROI was obtained by averaging the time courses across all voxels within the ROI. After generation of said BOLD fMRI time series, GSR was presented as an optional prepossessing step.

**Table 1:** The regions of interest in the study and their corresponding functional networks.

**#**	**Region of interest**	**Functional network**
1	Left amygdala	Limbic
2	Left anterior insula	Limbic
3	Left cuneus	Visual
4	Left frontal eye field	Dorsal attention
5	Left inferior frontal lobe	Dorsal Attention
6	Left inferior parietal lobe	Default mode
7	Left mid frontal gyrus	Dorsal Attention
8	Left parahippocampus	Limbic
9	Left posterior intraparietal sulcus	Dorsal attention
10	Left primary auditory cortex	Auditory
11	Left primary visual cortex	Visual
12	Left superior occipital lobe	Visual
13	Left superior temporal junction	Auditory/Limbic/Visual
14	Left superior temporal sulcus	Auditory
15	Left ventral intraparietal sulcus	Dorsal attention
16	Medial prefrontal cortex	Default mode
17	Posterior cingulate cortex	Default mode
18	Precuneus	Default mode
19	Right amygdala	Limbic
20	Right anterior insula	Limbic
21	Right cuneus	Visual
22	Right frontal eye field	Dorsal attention
23	Right inferior frontal lobe	Dorsal Attention
24	Right inferior parietal lobe	Default mode
25	Right mid frontal gyrus	Dorsal Attention
26	Right parahippocampus	Limbic
27	Right posterior intraparietal sulcus	Dorsal attention
28	Right primary auditory cortex	Auditory
29	Right primary visual cortex	Visual
30	Right superior occipital lobe	Visual
31	Right superior temporal junction	Auditory/Limbic/Visual
32	Right superior temporal sulcus	Auditory
33	Right ventral intraparietal sulcus	Dorsal attention

It has been argued that frequencies higher than 0.1 Hz do not contribute to resting state regional coherency as they are often related to physiological noise including cardiac and respiratory cycles (Cordes et al., 2001). As a result, the time series data were band-pass filtered, using a Bessel filter with low and high cutoff frequencies of 0.008 and 0.08 Hz as a baseline. Before filtering, the time series were mean centered, de-trended, and scaled. Three scaling methods were considered, scaling relative to: the quadratic variation, the norm, and the standard deviation of the time series. In the cyclicity analysis, the signals were also end-matched before scaling.

### 3.3. Features

After preprocessing, by employing seed-based connectivity approach, the fMRI time series of the 33 ROIs were analyzed using four rsFI techniques to generate 33 × 33 feature matrices. This resulted in:

1. Skew-symmetric lead matrices (LM), using cyclicity analysis,
2. Symmetric zero-lag correlation matrices (CM), using correlation analysis,
3. Symmetric lagged correlation matrices (LCM), using correlation analysis,
4. Symmetric dynamic time warping matrices (DM), using dynamic time warping algorithm.

### 3.4. Stability and Robustness

Similar to our previous work (Zimmerman et al., 2018), we evaluated the stability and reliability of rsFI features in identifying individuals by assessing the degree to which each rsFI feature remained invariant across the two visits. To do so, we trained 1-nearest neighbor classifiers (with cosine similarity) on scans from one of the two visits and tested on scans from the other visit and vice versa, resulting in running two classifiers. The accuracy was measured using the *R*_*K*_ correlation coefficient, which is a multi-class extension of the Mathews correlation coefficient for two class confusion matrices (Gorodkin, 2004). Further, to examine the robustness of each feature to GSR and filtering cutoff frequencies, the effect of these choices on the stability of each matrix was assessed. Additionally, for each subject and for each feature, we measured the cosine differences between features with and without GSR.

### 3.5. Classification

To evaluate the efficacy of each rsFI feature in identifying the neural correlates of tinnitus, we investigated the separability of tinnitus from controls by assessing the accuracy of three classifiers: (1) discriminant analysis, (2) support vector machines, and (3) convolutional neural networks. Discriminant analysis and support vector machine are classical methods in machine learning and we refer the reader to Theodoridis and Koutroumbas (2009) for the mathematical details and implementation. Convolutional neural network is a modern machine learning technique widely used to analyze visual patterns; for more details, see Krizhevsky et al. (2012). In the following subsections, we briefly introduce these classifiers and list the preprocessing steps specific to the classifier at hand. Monte Carlo cross validation with 100 times repetition was used to train and test all the classifiers. Seventy percent of the data were randomly extracted to train and the remainder of the dataset was used to test the classifiers. Discriminant analysis and support vector machine implementations found in Pedregosa et al. (2011) and neural network package described in Abadi et al. (2015) were used toward this purpose.

#### 3.5.1. Discriminant Analysis

Linear discriminant analysis (LDA) and quadratic discriminant analysis (QDA) are two widely-used classification techniques, which are based on Bayes classifier approximation. Both LDA and QDA assume that the probability density function of the input data (features, independent variables, predictors, etc.) for each class are drawn from a Gaussian distribution (James et al., 2013). LDA assumes that all Gaussian distributions have equal variance (in the case of univariate distributions) or covariance matrices (in the case of multivariate distributions) whereas QDA assumes each distribution has a distinctive variance or covariance matrix. The LDA decision boundary is a linear function of the inputs whereas QDA decision boundary is a quadratic function of the inputs, both estimating the Bayes decision boundary (James et al., 2013). In the case of multivariate distributions with a large number of dimensions, dimension reduction techniques are required to prevent numerical instability while calculating the inverse of the covariance matrices, especially, when the number of training samples is relatively small.

In our experimental setup, given that the feature matrices are either symmetric or skewed-symmetric, we only considered the upper triangular part of each feature matrix as the input data. Before dividing the data into the training and test sets, the data were vectorized and normalized. Due to the low number of training observations (i.e., number of unique subjects in the smallest group) relative to the input dimension (i.e., 528), principal component analysis (PCA) was applied to project the input data onto a 10-dimensional space before feeding the training data into the LDA or the QDA classifier and performing discriminant analyses.

#### 3.5.2. Support Vector Machine

In the past three decades, support vector machine (SVM) has emerged as one of the most popular classification techniques. Traditional SVM is a maximum margin classifier developed based on the linear classification problem, involving the construction of a separating hyperplane that breaks up training observations based on the corresponding class labels. The margin being defined as a distance between any two hyperplanes that obtain separation in the linearly separable case, SVMs solve an optimization problem with some specific constraints on finding an optimal hyperplane that maximizes the margin (James et al., 2013). The name SVM comes from the definition of the vectors that lie on the maximal margin as “support vectors.” With the introduction of the kernel trick (Boser et al., 1992), the constructed hyperplane can be linear or non-linear depending on the kernel used to transform the representation of the input data. In our experimental setup, the SVM with linear, quadratic, and Gaussian kernels were examined. To reduce over-fitting, slack variable and tuning parameter were used to allow misclassification of some training data close to margin.

Similar to DA, the upper triangular part of the feature matrices was vectorized. Following this, the data were standardized by removing the mean and scaling to unit variance ($Z=\frac{X-\mu }{\sigma }$, where μ and σ are empirical mean and standard deviation). Further, PCA was used to reduce the dimension of the input space from 528 to 20. Finally, the data were divided into training and test sets and fed into the SVM classifier.

#### 3.5.3. Convolutional Neural Networks

Using convolutional neural network (CNN), we examined the efficacy of rsFI features in distinguishing patients with tinnitus from those without tinnitus based on the visual interactivity patterns manifested in the feature matrices. CNN is a class of artificial neural networks that uses convolution operation inspired by visual neuroscience (Goodfellow et al., 2016). Contrary to the conventional neural networks, CNN introduces sparse connectivity by sharing parameters leading to fewer number of parameters. Similar to conventional neural networks, CNN can use gradient-descent based algorithms to classify training observations according to their class labels. Considering the small sample size in our experimental setting, CNN with simple architectures was assessed to reduce overfitting. A CNN was trained and tested on each feature matrix. The CNN architecture proposed by Meszlényi et al. (2017a) was adopted wherein they showed that CNN can differentiate between subject groups using rsFC data. The reader is referred to Figure S1 for a detailed explanation of the CNN architecture.

The actual power of the CNN classifier is manifested when trained with large datasets. Considering the small size of our database, in order to increase the efficacy of the CNN classifier, we used a generative model to generate synthetic samples, augmenting our training data. Particularly, variational auto-encoders (VAE) were implemented with two fully connected layers for both the encoder and decoder, and a two-dimensional latent space using mean-squared error loss as the construction loss and Kullback-Leibler divergence as the latent space loss. The key idea behind the VAE is to approximate the probability distribution of the input data in a latent space from which synthetic samples can be later drawn. For further details, see Kingma and Welling (2013) and Figure S2.

**Figure 1:**
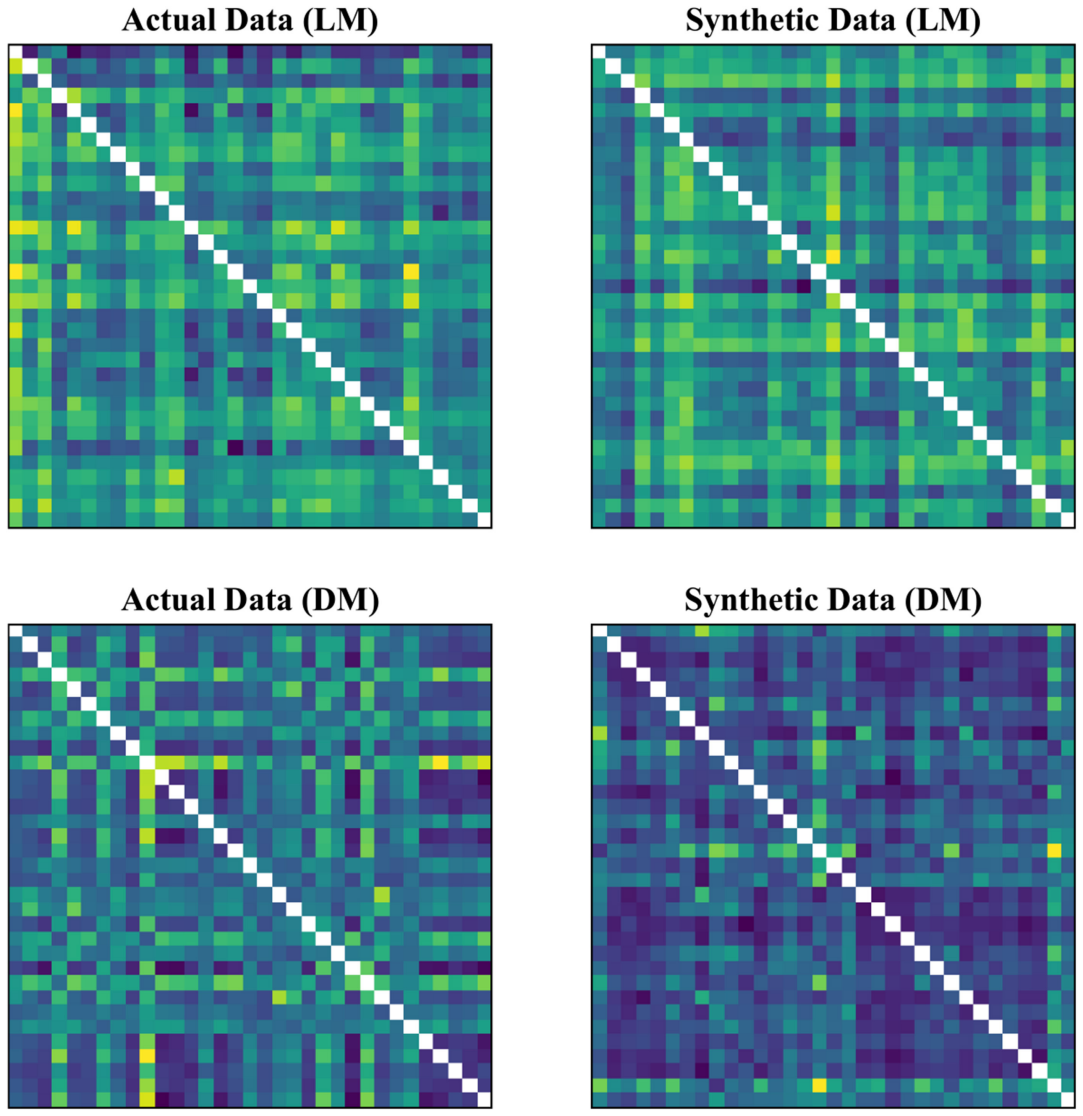
Generating synthetic data using variational auto-encoders. LM, lead matrix; DM, dynamic time warping distance matrix. Since the VAE trains to encode and decode on vectorized versions of the feature matrices (with redundant information removed), the visualization above is generated by vectors recast into matrix form. Therefore, the diagonal entries (which, indeed, are either zero or one) are irrelevant.

**Figure 2:**
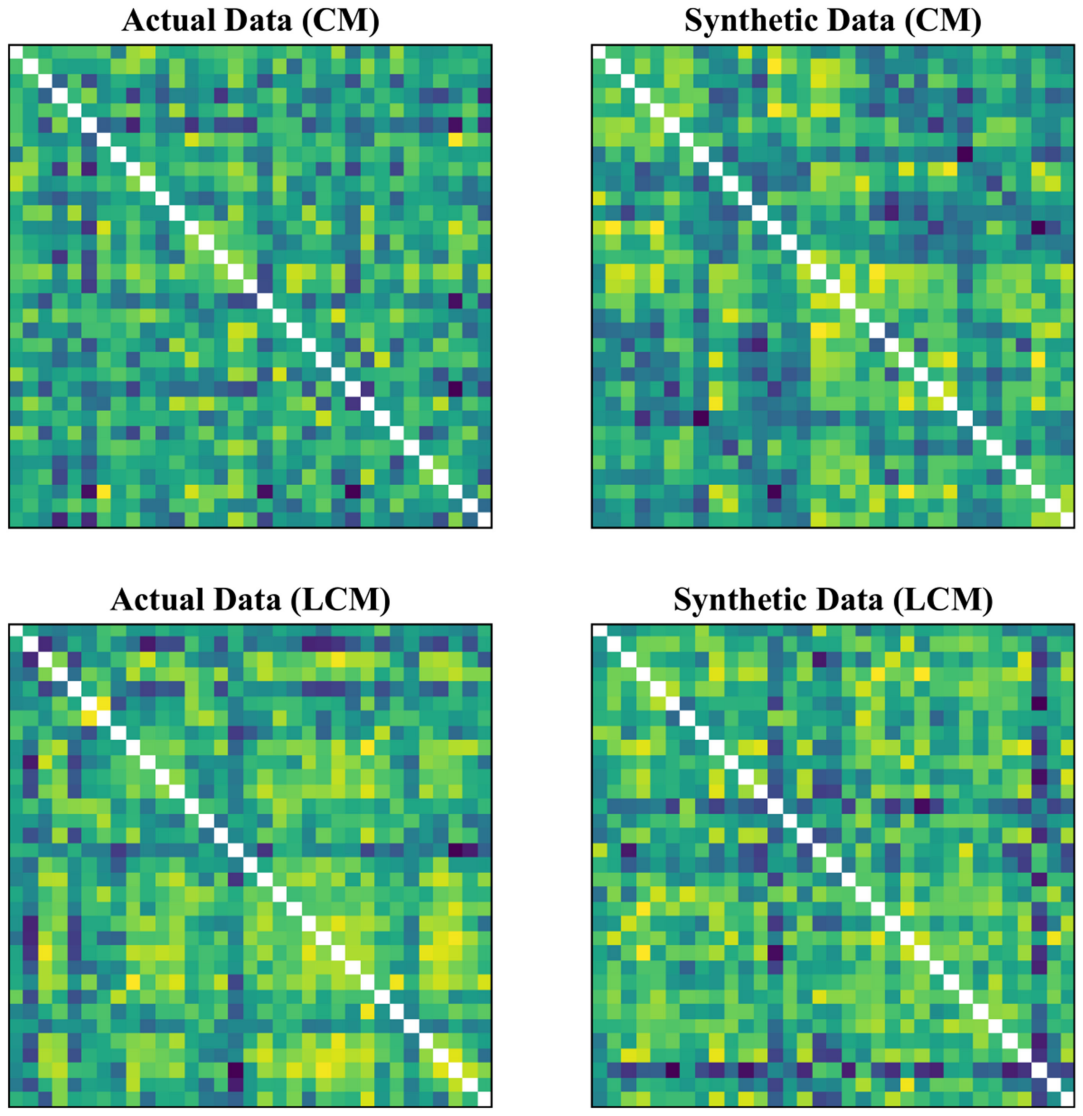
Generating synthetic data using variational auto-encoders. CM, zero-lag correlation matrix; LCM, lagged correlation matrix. Since the VAE trains to encode and decode on vectorized versions of the feature matrices (with redundant information removed), the visualization above is generated by vectors recast into matrix form. Therefore, the diagonal entries (which, indeed, are either zero or one) are irrelevant.

In our experimental setup, we used the data from both subject groups to train VAE. After training, no distinct separation between the patient and control distributions was found in the latent space. To overcome this, we trained VAE separately for each class. In doing so, the data were normalized and subsequently divided into the test and training sets. The data for each class were further normalized before training the VAE and later mean adjusted and re-scaled after training. Finally, we re-assessed the CNN performance using the augmented data. Figures 1, 2 demonstrate the synthetic LM, DM, CM, and LCM along with an example from their corresponding actual data. Note that for purposes of training the machine-learning models in this study (except for the CNN), the feature matrices were always vectorized and had redundant information (i.e., the diagonal values and the lower or upper half) removed from them. Therefore, the generated augmented samples were actually vectors, which were later matricized for visualization purposes or training the CNN; consequently the diagonal values in Figures 1, 2 hold no meaning.

## 4. Results

### 4.1. Stability and Robustness

Tables 2, 3 summarize the accuracy of 1-nearest neighbor classifiers in identifying individuals, using feature matrices (LM, CM, LCM, and DM) with different filter settings and with & without GSR. The results showed that the extent to which feature matrices remained stable across the visits varied depending on the employment of GSR and the filters. In general, GSR improved the stability of all feature matrices except those generated by the DTW distance measure (i.e., DM). However, the effect of band-pass filtering was dependent on the feature matrices. Particularly, applying band-pass filters reduced the stability of LM regardless of employing GSR, whereas it increased the stability of other matrices, especially when combined with GSR. For all feature matrices, as the upper cutoff frequency of the band-pass filter increased from 0.08 to 0.2 Hz, stability was enhanced. The scaling method did not affect the general trend of stability except for DM, i.e., scaling the time series using either the norms or standard deviation adversely affected the stability of DM. For simplicity, only the results using quadratic scaling are presented here and those related to other scaling methods are given in Tables S1, S2.

**Table 2:** The accuracy rate of 1-nearest neighbor classifier to identify individuals across two scan sessions held 1 week apart with different filter specifications and no GSR.

	**No GSR**		
**Feature**	**No filter**	**BPF (**0.008 ≤ *f*_*pass*_ ≤ 0.08** Hz)**	**BPF (**0.008 ≤ *f*_*pass*_ ≤ 0.2** Hz)**
LM	0.68	0.45	0.67
CM	0.53	0.53	0.69
LCM	0.48	0.45	0.64
DM	0.32	0.47	0.56

*The time series were normalized using their quadratic variation. LM, Lead Matrices; CM, Zero-lag Correlation Matrices; LCM, Lagged Correlation Matrices; DM, Dynamic Time Warping Matrices; BPF, Band-Pass Filter; GSR, Global Signal Regression*.

**Table 3:** The accuracy rate of 1-nearest neighbor classifier to identify individuals across two scan sessions held 1 week apart with different filter specifications and GSR.

	**With GSR**		
**Feature**	**No filter**	**BPF (**0.008 ≤ *f*_*pass*_ ≤ 0.08** Hz)**	**BPF (**0.008 ≤ *f*_*pass*_ ≤ 0.2** Hz)**
LM	0.83	0.56	0.79
CM	0.78	0.82	0.92
LCM	0.76	0.80	0.90
DM	0.28	0.42	0.53

*The time series were normalized using their quadratic variation. LM, Lead Matrices; CM, Zero-lag Correlation Matrices; LCM, Lagged Correlation Matrices; DM, Dynamic Time Warping Matrices; BPF, Band-Pass Filter; GSR, Global Signal Regression*.

**Figure 3:**
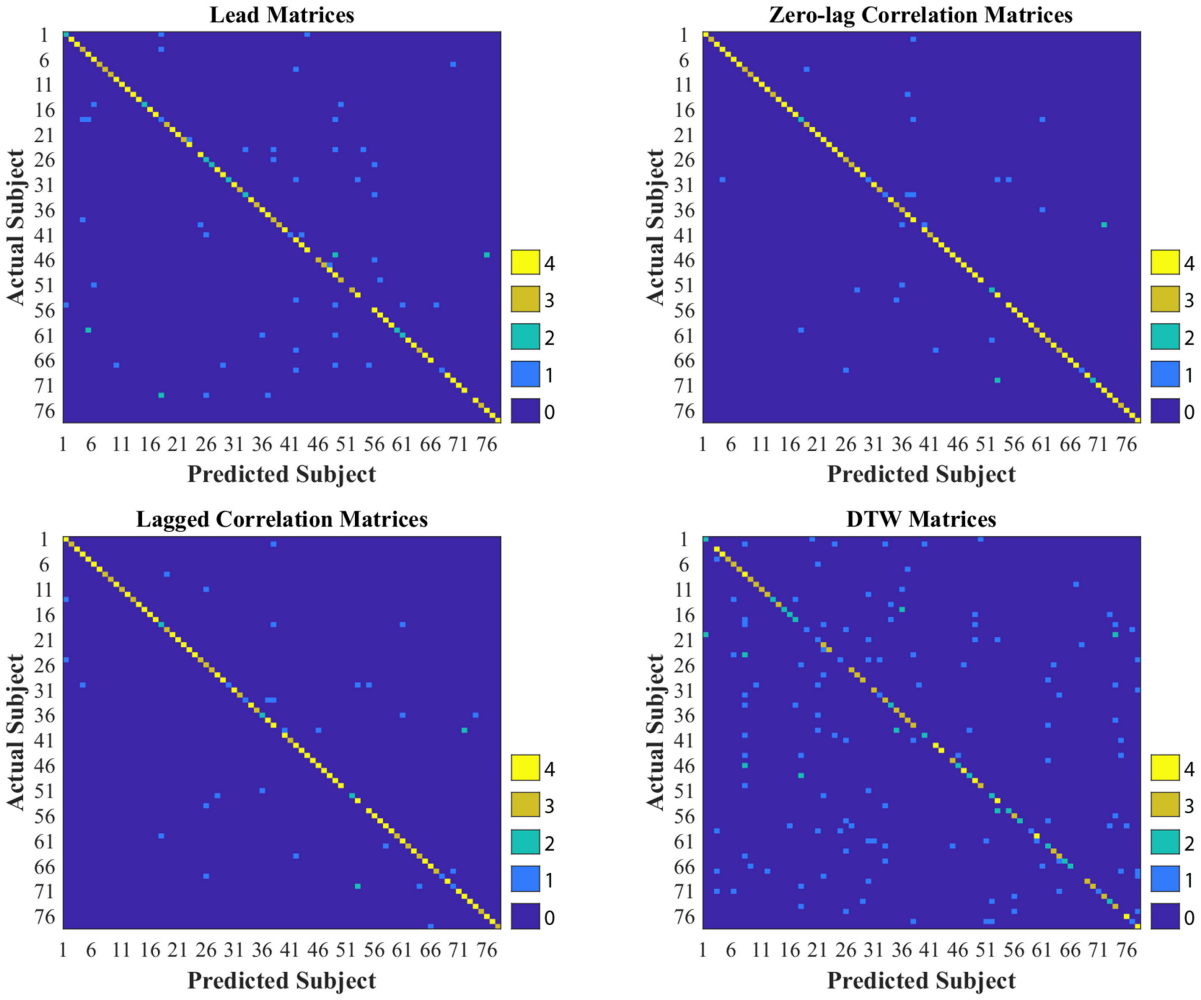
Confusion matrices of 1-nearest neighbor classifiers with the highest accuracy for each interactivity feature set used to identify individuals across two visits, held 1 week apart. DTW stands for dynamic time warping.

As shown in Tables 2, 3, CM had the highest stability, and DM had the lowest stability across the visits. Specifically, both CM and LCM had the highest stability (92 and 90%, respectively) when GSR was applied with band-pass filtering (upper cutoff frequency of 0.2 Hz). LM also showed the highest stability (83%) when the global signal was regressed, but with no filtering. Like CM and LCM, applying band-pass filters, specifically with the upper cutoff frequency of 0.2 Hz, increased the stability of DM. However, unlike the other feature matrices, DM demonstrated the highest stability (0.56%) when the global signal was not regressed. Figure 3 depicts the confusion matrices of 1-nearest neighbor classifiers for the conditions in which each feature matrix exhibited the highest reliability.

**Figure 4:**
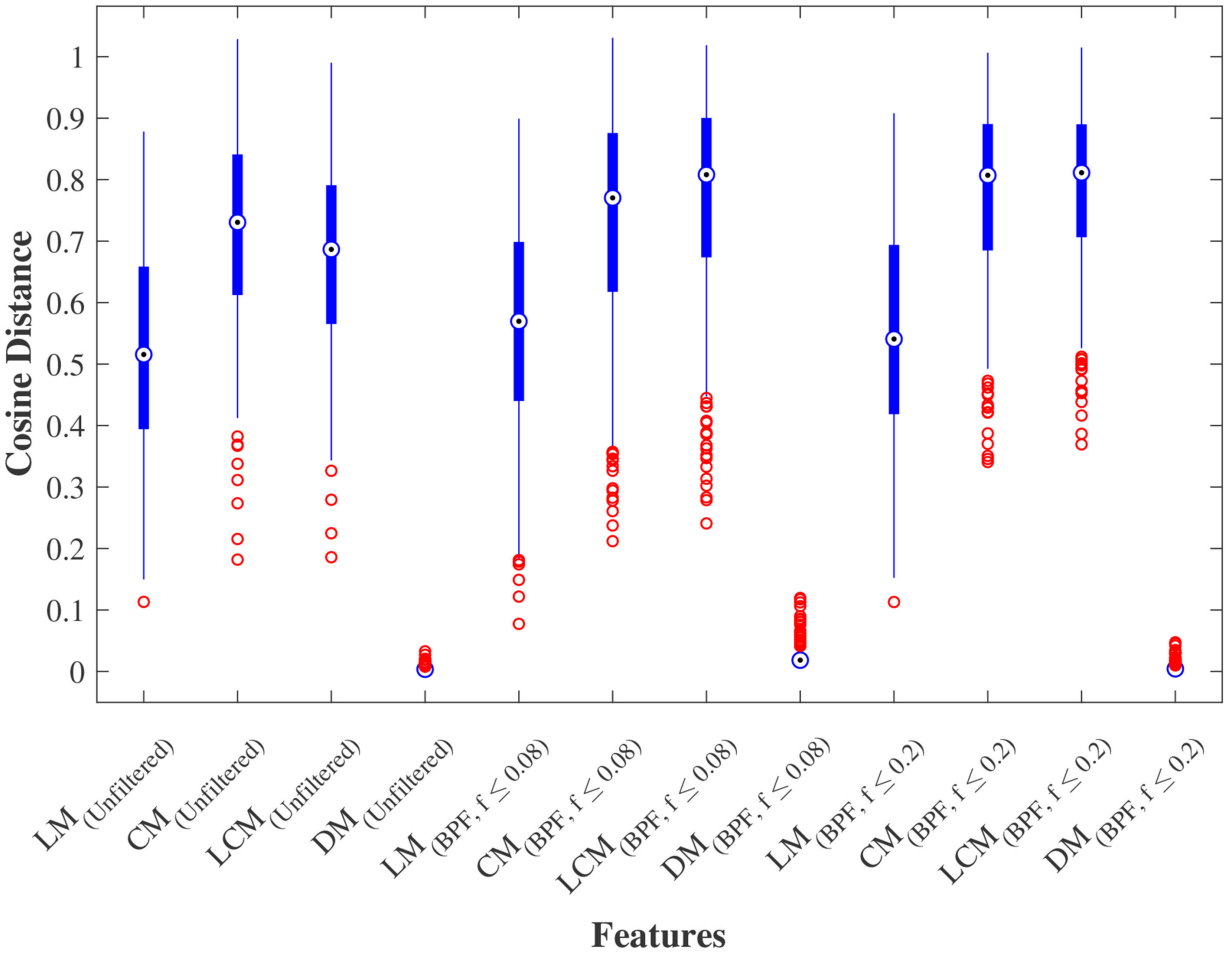
Robustness to global signal regression. The distribution of the cosine distances between with and without global signal regression across all subjects for each feature under three conditions: (1) No filtering, (2) Band-pass filtering (0.008 ≤ *f*_*pass*_ ≤ 0.08 Hz), and (3) Band-pass filtering (0.008 ≤ *f*_*pass*_ ≤ 0.2 Hz). LM, CM, LCM, and DM stand for lead matrix, zero-lag correlation matrix, lagged correlation matrix, and dynamic time warping matrix, respectively.

We further examined the robustness to GSR, by measuring the cosine distance between the feature matrix with GSR and the feature matrix without GSR for each individual subject. Figure 4 shows the distribution of these distances measured for LM, CM, LCM, and DM, under three filtering conditions. A non-parametric Kruskal-Wallis test (followed by a Mann-Whitney U *post-hoc* test with Bonferroni correction) revealed that DM was the feature matrix most robust to GSR, whereas CM and LCM were the feature matrices least robust to GSR. The statistical test also showed that band-pass filtering significantly reduced the robustness to GSR, for all feature matrices.

### 4.2. Classification

Overall, the performance of all classifiers was close to or slightly better than chance, indicating a poor dissociation between tinnitus patients and healthy controls. Nevertheless, there were some differences across classifiers and input feature matrices. That is, the performance of SVM on LCM (sensitivity of 64% and specificity of 54%) surpassed the performance of all other classifiers. Also, incorporating augmented data improved the sensitivity of the CNN on all feature matrices, but deteriorated the specificity, except for DM. Specifically, using the condition in which DM had the worst reliability in identifying individuals (i.e., with GSR and no filtering), the CNN had the best sensitivity (58%) and specificity (58%) in separating subject groups. This points to a trade-off between identifying at the individual level and generalizing to the group (Finn et al., 2017).

#### 4.2.1. Sparse SVM

Sparse linear SVM (Bi et al., 2003) was used to identify inter-regional functional interactions that played a dominant role in correctly separating tinnitus patients from controls. Subsequently, the classification accuracy of QDA, SVM, and CNN was re-evaluated using such selected variables. The standard risk function minimized by SVM includes two terms: the hinge loss function and squared ℓ_2_-norm of the weight vector. In sparse linear SVM, the ℓ_1_-norm is used as the regularization term in lieu of ℓ_2_-norm allowing for a sparser solution of the weight vector. The non-zero elements of the weight vector then inherently correspond to variables that play more dominant roles in building the optimal separating hyperplane.

**Figure 5:**
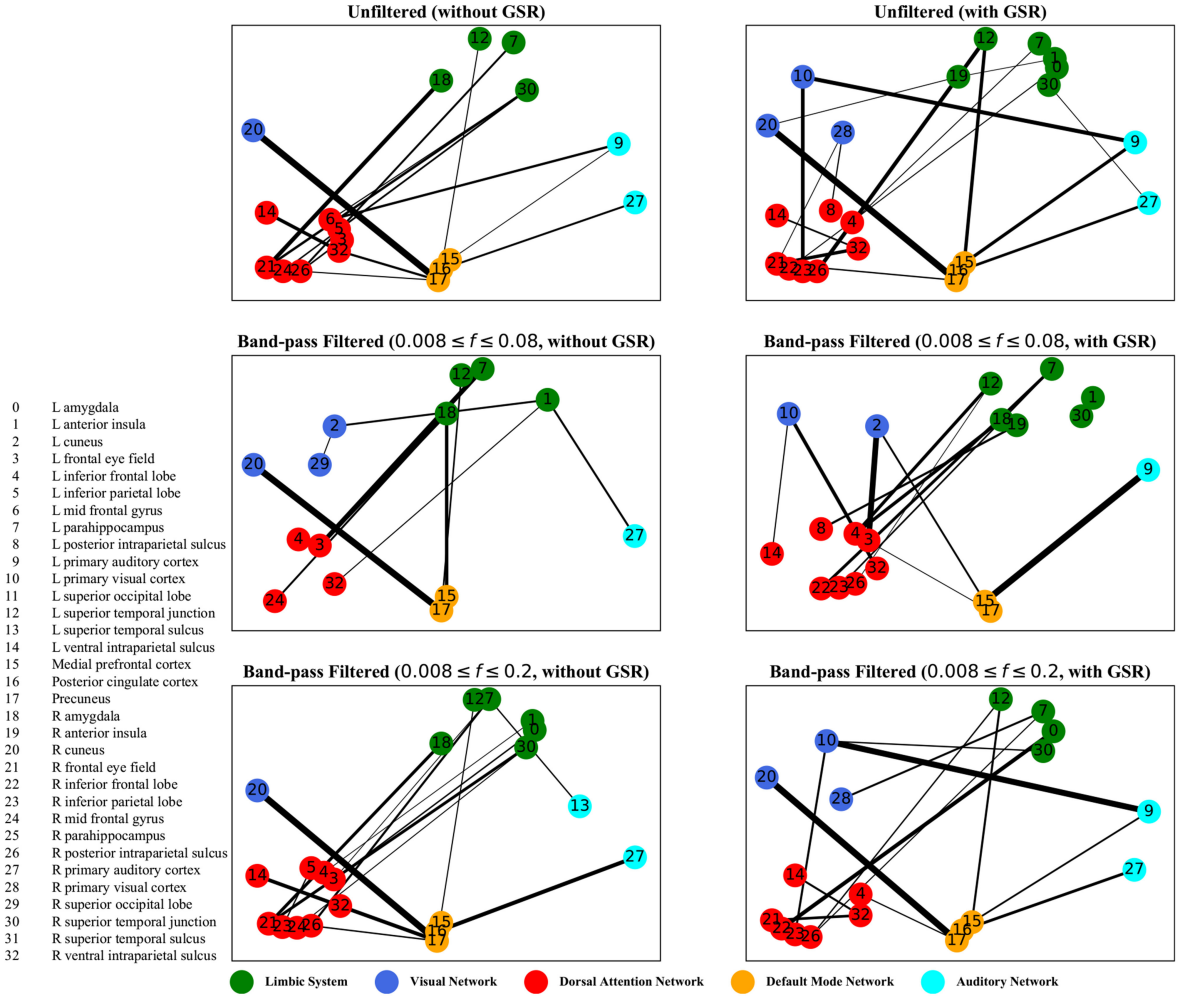
Cyclicity analysis—the salient interactions in separating patients and control groups, selected by sparse SVM. The nodes correspond to the ROIs listed on the left side of the figure. The edges represents the interaction between ROIs corresponding to SVM solution of the weight vector. The width of the weights relates to the magnitude of interactions. GSR stands for global signal regression and *f* denotes the passed frequencies of band-pass filters.

**Figure 6:**
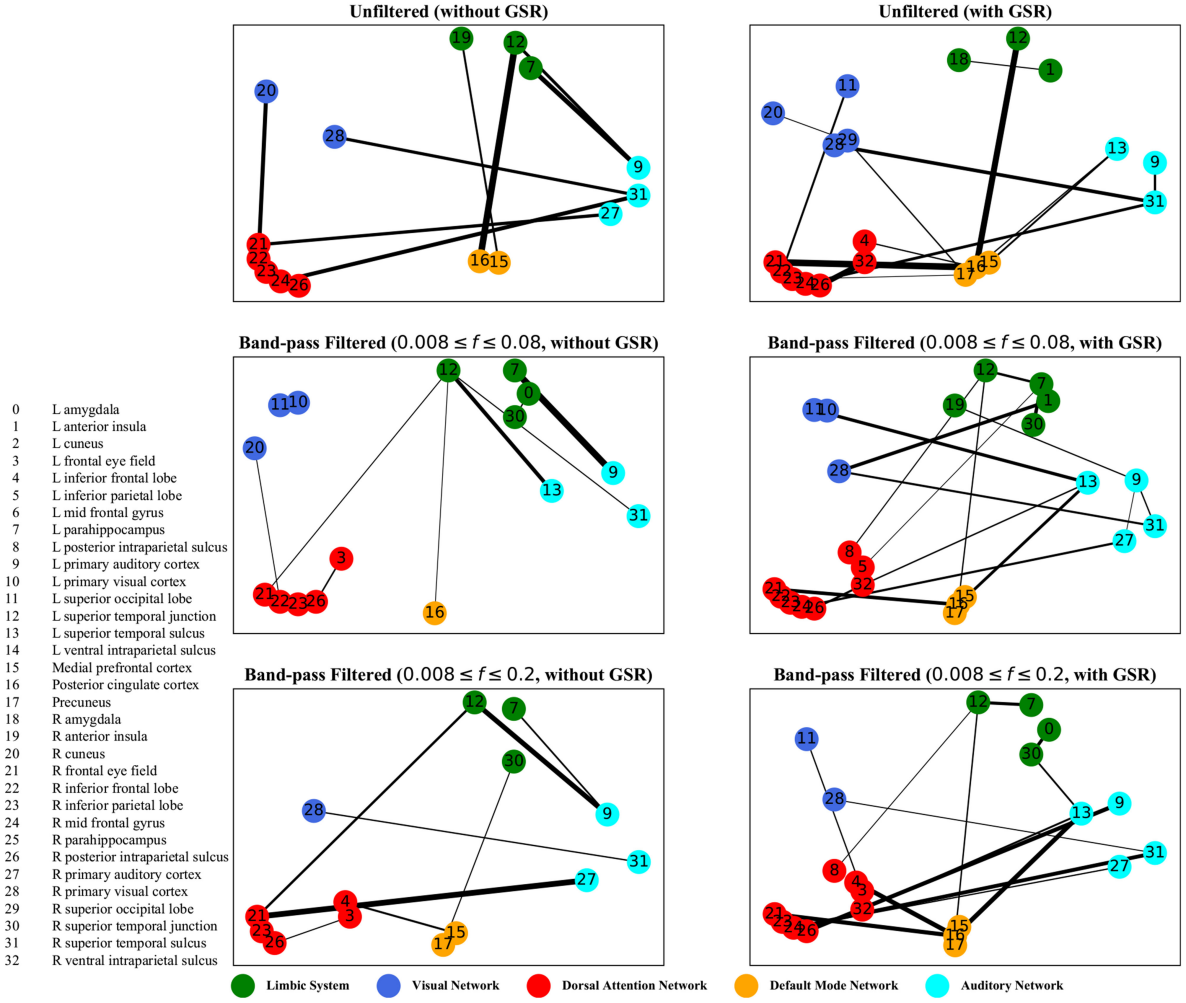
Zero-lag correlation analysis—the salient interactions in separating patients and control groups, selected by sparse SVM. The nodes correspond to the ROIs listed on the left side of the figure. The edges represents the interaction between ROIs corresponding to SVM solution of the weight vector. The width of the weights relates to the magnitude of interactions. GSR stands for global signal regression and *f* denotes the passed frequencies of band-pass filters.

**Figure 7:**
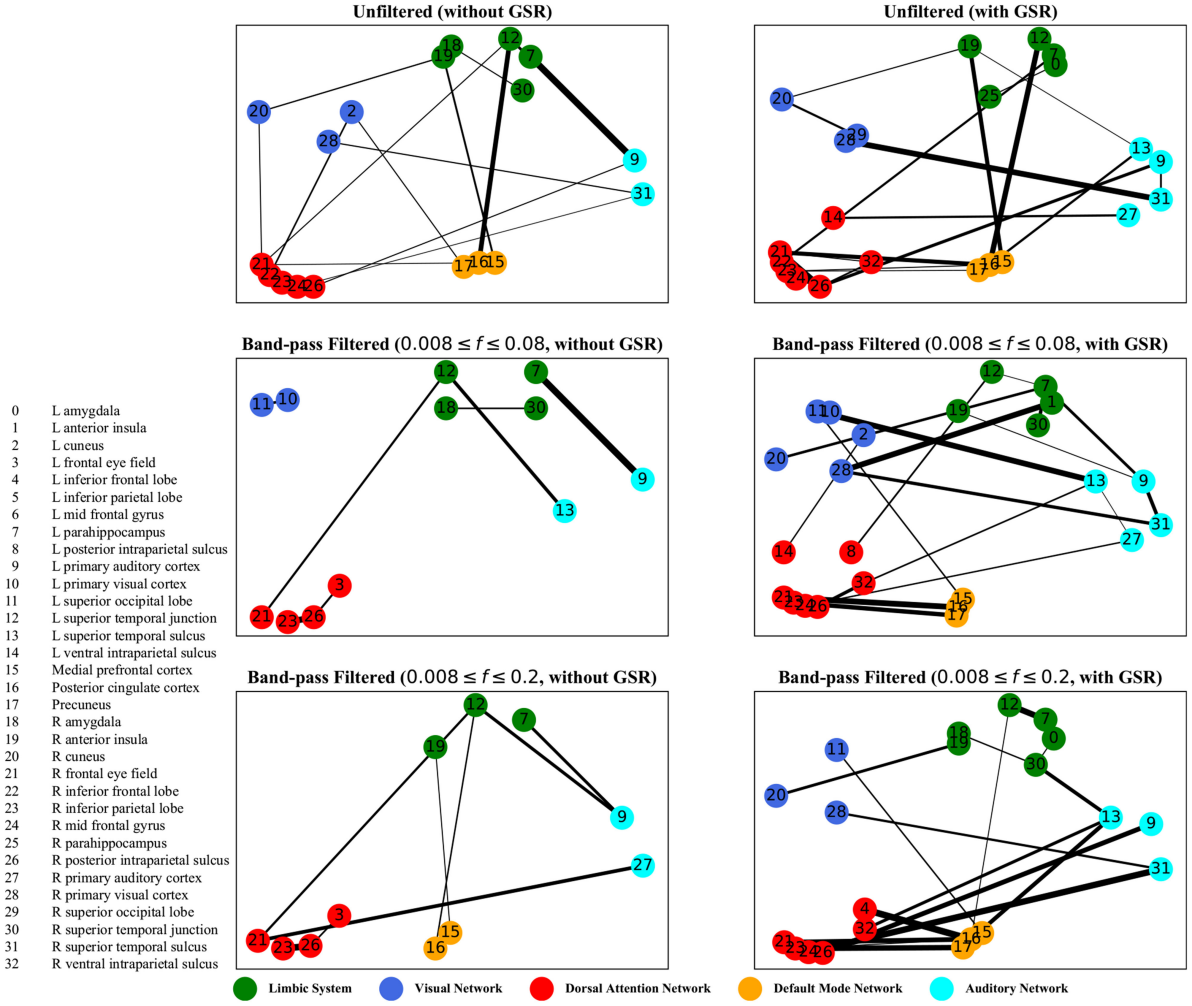
Lagged correlation analysis—the salient interactions in separating patients and control groups, selected by sparse SVM using lagged matrices. The nodes correspond to the ROIs listed on the left side of the figure. The edges represents the interaction between ROIs corresponding to SVM solution of the weight vector. The width of the weights relates to the magnitude of interactions. GSR stands for global signal regression and *f* denotes the passed frequencies of band-pass filters.

**Figure 8:**
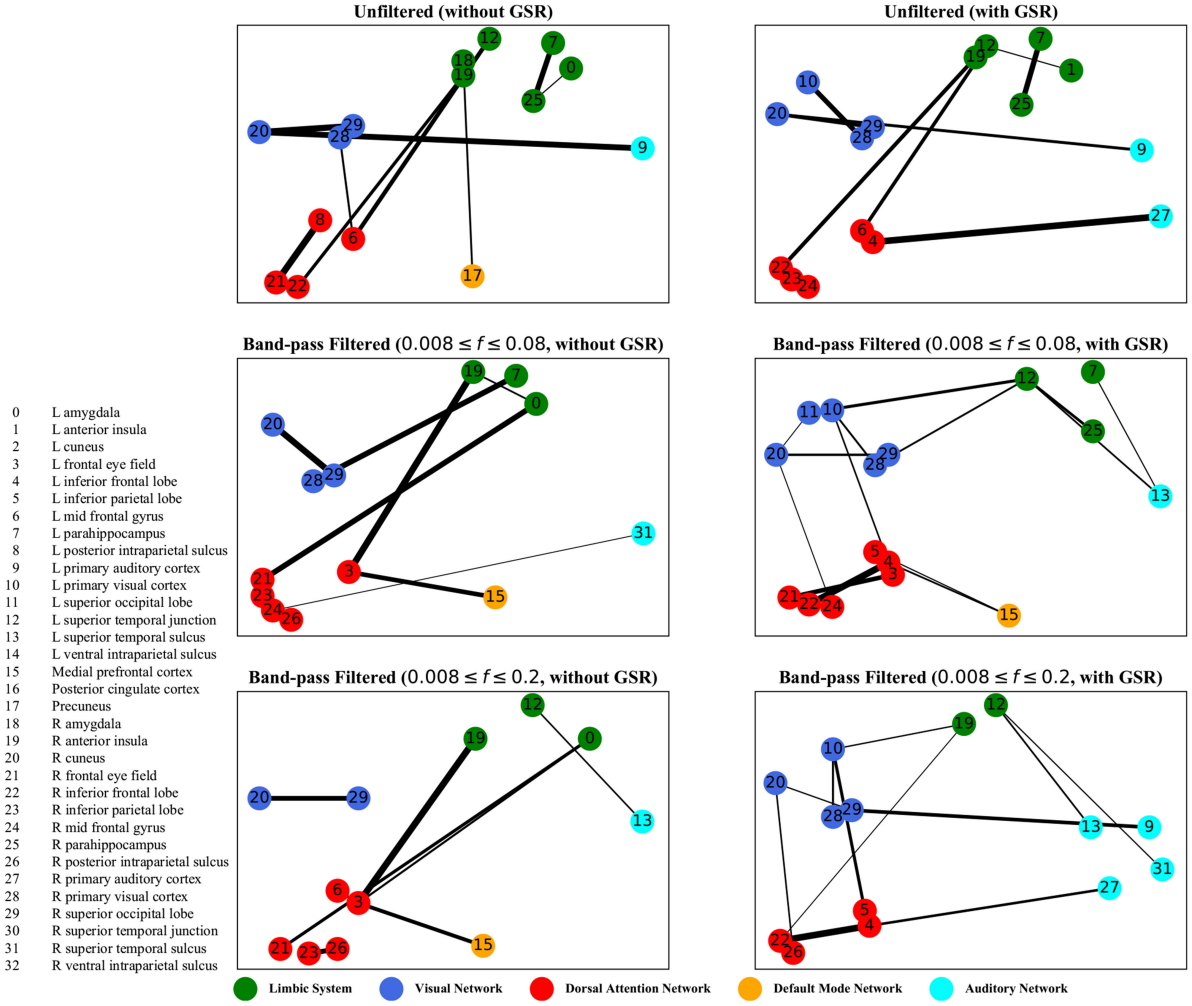
Dynamic time warping distance—the salient interactions in separating patients and control groups, selected by sparse SVM using dynamic time warping matrices. The nodes correspond to the ROIs listed on the left side of the figure. The edges represents the interaction between ROIs corresponding to SVM solution of the weight vector. The width of the weights relates to the magnitude of interactions. GSR stands for global signal regression and *f* denotes the passed frequencies of band-pass filters.

The graphs shown in Figures 5–8 depict the salient inter-regional interactions as quantified by cyclicity analysis, zero-lag correlation, lagged correlation, and DTW distance measure, respectively. Each ROI is represented by a node and the interaction between ROIs is depicted as an edge. The width of each edge signifies the magnitude of the interaction, which corresponds to the absolute values of the weight vector obtained in the SVM solution. Using these dominant interactions, the dimension of each feature matrix was reduced and used to re-evaluate the classifiers. The results showed that dimensionality reduction based on sparse SVM improved the classification. Again, SVM (here, on CM instead of LCM) outperformed other classifiers, with sensitivity and specificity of 62%.

As illustrated in Figures 5–8, sparse SVMs found different salient interactions between ROIs depending on the rsFI feature matrix, filtering setting, and GSR. For example, using LM without filtering and without GSR, the sparse SVM found an interaction between precuneus and the right cuneus as a dominant interaction in separating tinnitus from controls. In addition to the aforementioned interactivity, using LM with filtering (upper cutoff frequency of 0.2 Hz) and with GSR, the sparse SVM found the interactivity between the left primary visual cortex and the left primary auditory to be useful in separating subject groups. For all matrices except DM, using GSR decreased the degree of sparsity obtained in the solution, leading to the hyperplanes defined by more non-zero elements when differentiating subject groups. Here, the robustness of DM to GSR is consistent with the results observed in the stability analysis, where DM was the feature matrix most robust to GSR. In sum, the importance of interactions between different ROIs in building the optimal separating hyperplane was found to depend on the particular feature matrix and chosen preprocessing steps.

#### 4.2.2. Non-linear Dimensionality Reduction

**Figure 9:**
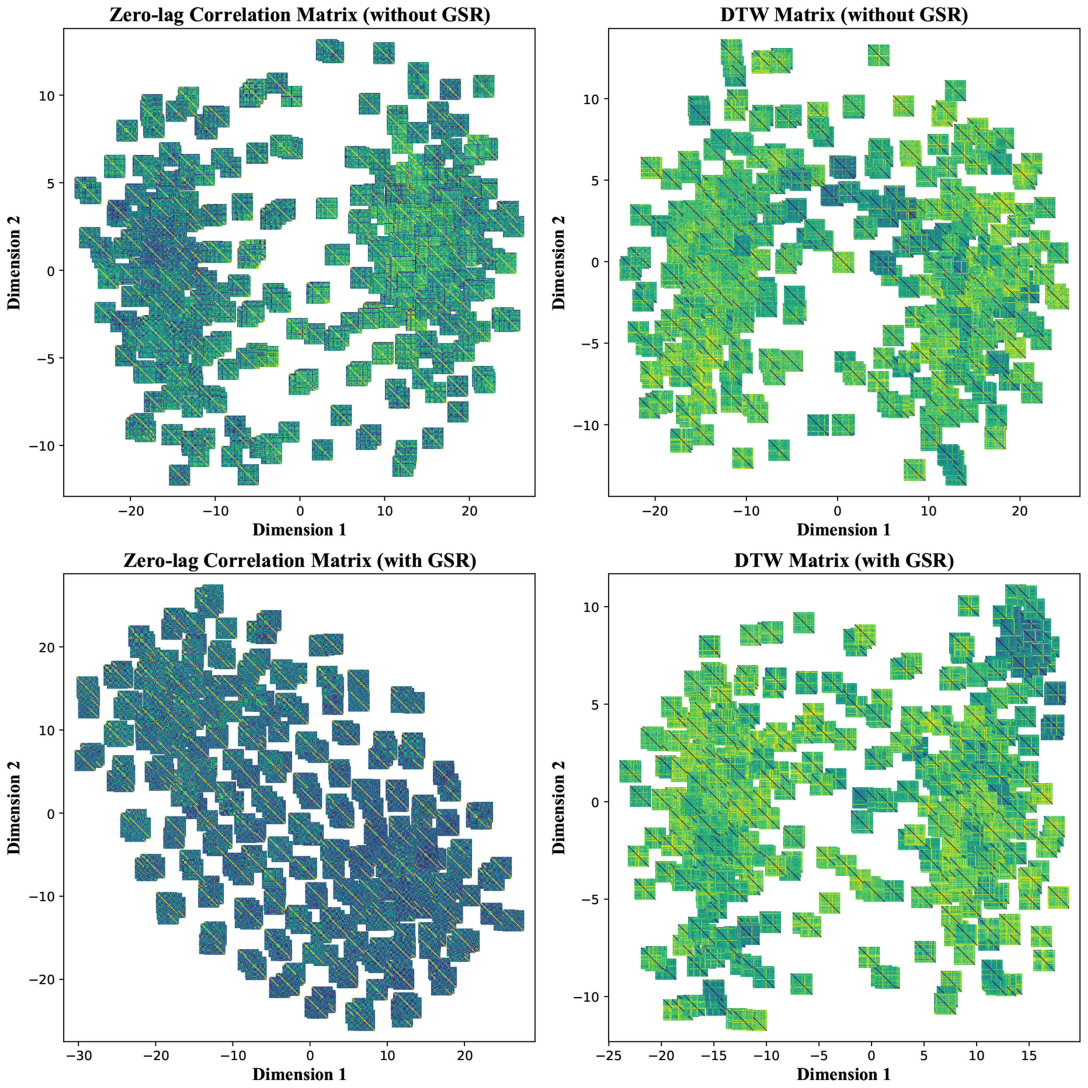
T-distributed Stochastic Neighbor Embedding (t-SNE)—Non-linear dimensionality reduction to two dimensions of the zero-lag correlation and dynamic time warping (DTW) matrices without and with global signal regression (GSR). The resting state fMRI data were band-pass filtered (0.008 ≤ *f*_*pass*_ ≤ 0.2) before extracting the features. Each observation is depicted with its corresponding feature matrix.

**Figure 10:**
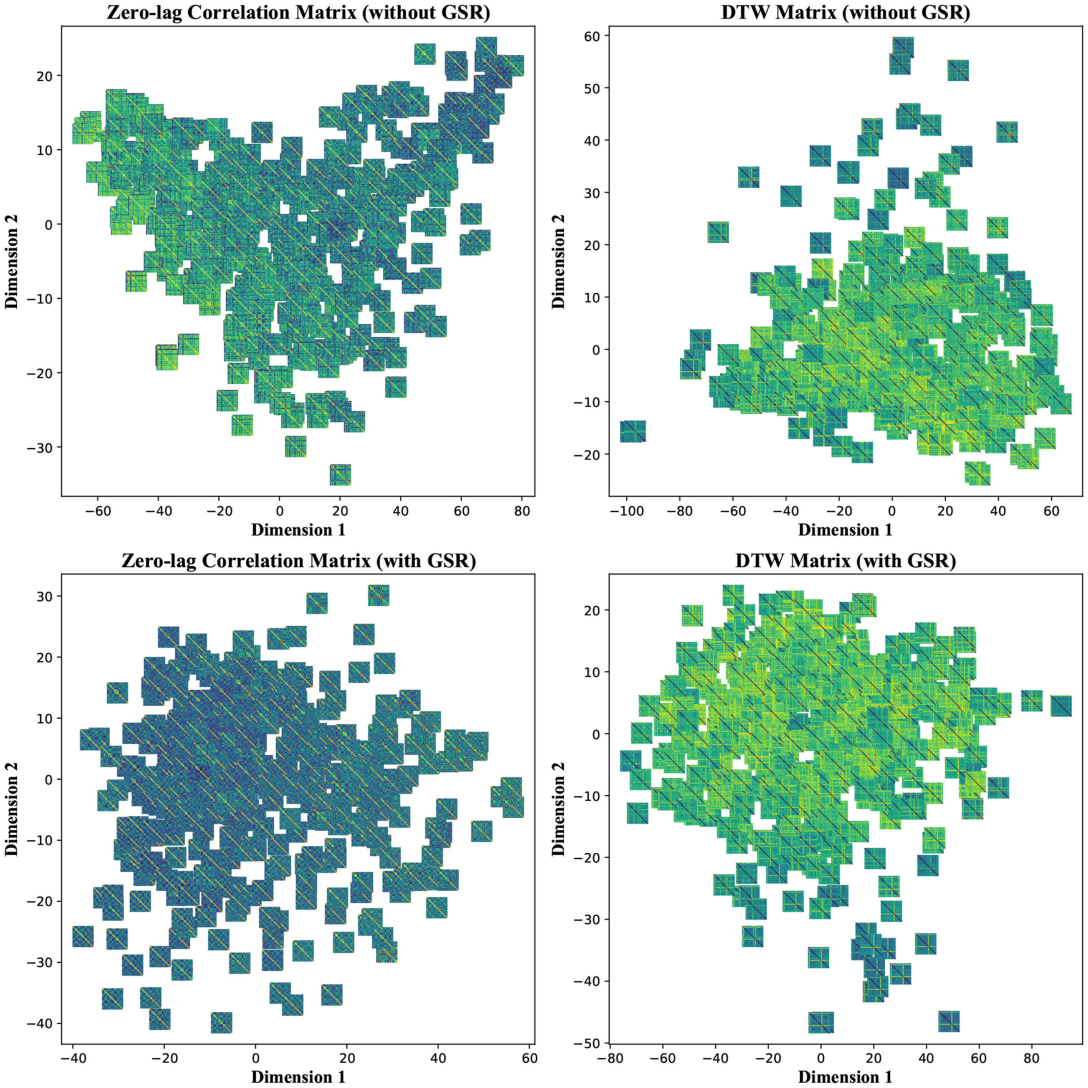
Isomap—Non-linear dimensionality reduction to two dimensions of the zero-lag correlation and dynamic time warping (DTW) matrices without and with global signal regression (GSR). The resting state fMRI data were band-pass filtered (0.008 ≤ *f*_*pass*_ ≤ 0.2) before extracting the features. Each observation is depicted with its corresponding feature matrix.

To further investigate the poor performance in group separability, we visualized each feature matrix across all participants using t-distributed stochastic neighbor embedding (t-SNE), which is a non-linear dimensionality reduction method (Maaten and Hinton, 2008). Using t-SNE with perplexity (a hyper parameter in the algorithm) of 50, CM, LCM, and DM were divided into two distinct clusters after mapping into a 2-dimensional space. Such clear separation was less pronounced in the case of LM, regardless of GSR. GSR, no matter the filter setting, changed both CM and LCM into a continuum and obscured the boundary between the two clusters. This was not the case with DM, which remained as two clusters. This is an observation consistent with previous stability and classification results, which underscores the robustness of DM to GSR. Figure 9 depicts CM and DM of both tinnitus and control populations after applying t-SNE, using band-pass filtering (0.008 ≤ *f*_*pass*_ ≤ 0.2) and with & without GSR. Each observation is shown with its corresponding feature matrix. Further investigation revealed that this observed clustering was not correlated with the subject group labels or any other metadata, such as hearing status, age, or gender. Nevertheless, as demonstrated in the upper left panel of Figure 9, it appears that the samples in one cluster have higher correlation values (illustrated by lighter colors) than the samples in another cluster. Using another popular non-linear dimensionality reduction method, Isomap (Tenenbaum et al., 2000), a trend similar to tSNE was observed. As illustrated in the upper left panel of Figure 10, the samples with greater values of Dimension 1, depicted by darker colors in the right, can be distinguished from those with smaller values in the left. Like tSNE, using GSR eliminated this differentiation in zero-lag correlation analysis, whereas it did not change such differentiation in DTW distance measures. The results of these visualization methods may help partially explain the low sensitivity and specificity observed in the classification results. Though both stochastic and deterministic unsupervised clustering methods found a natural separation into low and high values in some two-dimensional latent space, the obtained separation was not materially related to the group labels at hand. Examples of the algorithms applied to other feature matrices can be found in Figures S3, S4.

## 5. Discussion

Altogether, the results of this study indicate that the surveyed fMRI rsFI methods, including cyclicity analysis and pre-established functional connectivity methods, generate features that convey sufficient information for identifying individuals across visits on different days. However, these methods may poorly manifest the group-level information essential to reliably identify tinnitus population investigated in our study.

### 5.1. Identifying Subject Groups

As an example patient group, in this study we focused on tinnitus. In clinical settings, tinnitus is commonly diagnosed by self-report because there are no diagnostic tools available to objectively identify this condition. Using brain imaging, specifically fMRI, previous studies including our own work have reported intrinsic large-scale neural networks implicated in tinnitus (Burton et al., 2012; Maudoux et al., 2012; Schmidt et al., 2013, 2017; Davies et al., 2014). Default mode (Raichle et al., 2001), dorsal attention (Fox et al., 2006), and auditory networks are examples of such neural ensembles. For a review, refer to Shahsavarani et al. (2019).

Identifying invariant neural correlates of tinnitus as objective neuroimaging measures can potentially advance the development of diagnostic and treatment tools for this disorder. Thus, it is imperative to evaluate the efficacy of the rsFI measures in dissociating patient groups from neurotypical controls. In the current study, we evaluated the effectiveness of machine-learning techniques (including DA, SVM, and CNN) in separating tinnitus from healthy groups using features extracted by cyclicity analysis, zero-lag correlation analysis, lagged correlation analysis, and dynamic time warping distance measure. Our experiments revealed a poor performance for the classifiers in identifying the two subject groups. Although reducing the number of dimensions (using sparse SVM) or augmenting the training data (for the CNN classifier) improved the performance of the classifiers, the classification problem still needs further enhancements to be competitive with results in similar approaches, for example, to cancer detection, susceptibility & prognosis studies etc. (Tan and Gilbert, 2003; Cruz and Wishart, 2007; Kourou et al., 2015).

Exploring the separability of subject groups, sparse SVM found different salient interactions between regions and networks in building a separating hyperplane to dissociate tinnitus from control, depending on the rsFI analysis. Recall that in the hinge loss:

(3) $$\begin{array}{r} l\left(y\right)=\max\left(0,1-ty\right)=\max\left(0,1-t\left(w\cdot x+b\right)\right), \end{array}$$

where *t* is the true label and *y* is the prediction, the elements of the vector *w* determine the optimal separating hyperplane (which maximizes the margin). In other words, the hinge-loss will choose a combination of the ROI interactions that form a maximum margin separating hyperplane between the two sets of labels because each element of our vector corresponds to the interaction between a pair of ROIs. However, in the sparse SVM formulation, the hinge loss is used in conjunction with a ℓ_1_ norm on *w*. This causes the optimization to have a trade-off between maximizing margin while maintaining classification accuracy and sparsifying *w*. Thus, it selects those ROI interaction pairs that determine the cardinal orientation of the hyperplane (to maximize accuracy) while reducing the number of components (by sacrificing on the margin), and thereby gives us interaction pairs that play a dominant role in differentiating the two classes. In our study, we found that different rsFI analyses resulted in different dominant ROI pair interactions in creating a separating hyperplane for the point cloud. For simplicity, here, we just discuss the condition in which the resting state fMRI time series were band-passed using the conventional upper cutoff frequency of 0.08 Hz, and without removing the global signal, as depicted in the left middle panels in Figures 5–8.

For cyclicity analysis, the interaction between precuneus and visual cortex as well as the interaction between the left parahippocampus and the left frontal eye field was found to contribute to the separation of tinnitus and control groups. Precuneus is a major hub in the default mode network and its interaction with other networks indicates that the coherency of the default mode network is disrupted in tinnitus. In fact, several studies have reported tinnitus-related decrease in the coherency of the default mode network (Schmidt et al., 2013; Carpenter-Thompson et al., 2015; Lanting et al., 2016; Leaver et al., 2016b). Given that the participants had their eyes open during the scan, the interaction between the default mode network and visual network is expected. The interesting point is that this interaction was different between the tinnitus and control groups. Parahippocampus is a part of the limbic system and the frontal eye field is a major node in the dorsal attention network. In a tinnitus model based on cognitive control of emotion (Husain, 2016), we suggest that the constant percept of tinnitus may correlate with changes in the interaction between the dorsal attention network and the limbic system, regulating the emotional response to tinnitus. The interaction between the parahippocampus and the frontal eye field found by sparse SVM is in support of this model.

For both zero-lag correlation and lagged correlation analyses, the interaction between the auditory network and the limbic system was predominant in separating subject groups. The central auditory regions are neuroanatomically connected to the limbic system, which evokes emotional responses to auditory stimuli. In turn, the limbic system interacts with the auditory system, which regulates sound perception based on emotional processing. Previous studies have reported on tinnitus-related alternations in the interaction between the auditory network and the limbic system (Rauschecker et al., 2010; Maudoux et al., 2012; Schmidt et al., 2013, 2017; Leaver et al., 2016a), which endorses the importance of this interaction found by sparse SVM.

For dynamic time warping distance measure, similar to cyclicity analysis, the interaction between the limbic system and the dorsal attention network was found to be different between the tinnitus and healthy control groups. In addition, the interaction between the left frontal eye field and the medial prefrontal cortex, part of the default mode network, was useful in differentiating tinnitus patients from healthy controls. Previous research from our lab (Schmidt et al., 2017) has shown that tinnitus is correlated with increased functional interactivity between the default mode network and the dorsal attention network, supporting the interaction between medial prefrontal cortex and the frontal eye field.

Although, the results from sparse SVM are consistent with the findings from previous studies, it is worth noting that the ROIs used in this study were chosen based on seminal work on tinnitus. Therefore, finding an interaction implicated in tinnitus is not surprising. In addition, the importance of the interactions in separating subject groups highly depended on the rsFI analysis method and the choice of preprocessing. This sensitivity to different methodologies and data analyses may help partly explain the inconsistent evidence provided by previous rsFC studies on tinnitus (Schmidt et al., 2017; Shahsavarani et al., 2019).

### 5.2. Identifying Individuals

The rsFI analyses explored in this study (i.e., cyclicity analysis, zero-lag correlation analysis, lagged correlation analysis, and dynamic time warping distance measure) extract distinctive or overlapping information from resting state fMRI data. Using data from patients with tinnitus and healthy controls, we found that the features generated by correlation-based methods and cyclicity analyses were highly reliable and reproducible across two visits (being 1 week apart). Our results also showed that employing GSR boosted the reliability of these features to identify individuals. This suggests that applying GSR may augment the representation of information at the level of individual participants.

Contrary to cyclicity and correlation-based analyses, the features generated by the DTW distance measures demonstrated poor reliability across visits, indicating that these features represent less participant-specific information compared with other techniques. This is in accordance with the observation in our study that using GSR did not alter the reliability of these features, implying the high robustness of the DTW distance measures to GSR. This finding is also consistent with the results presented by Meszlényi et al. (2017b) where they showed that both zero-lag and lagged correlation were less robust to GSR compared with DTW distance. Meszlényi et al. (2017b) also showed that using resting state features extracted by measuring the DTW distance between fMRI time series improved the accuracy of identifying subject groups using machine-learning techniques. As mentioned earlier, our results showed that the DTW distance was the least stable feature in identifying specific individuals across visits. Although we did not observe any advantage in using the DTW distance measures to classify our particular subject groups (except for CNN with augmented data), the findings across our study and Meszlényi et al. (2017b)'s study suggest that this measure may represent group-level information to a greater extent than the participant-specific information compared with other measures; a hypothesis that can be further investigated in future studies.

In general, band-pass filtering of the resting state fMRI time series deteriorated the reliability of cyclicity analysis, but either did not change or even improved the reliability of the features extracted by other methods. Especially, using the upper cutoff frequency of 0.2 Hz increased the accuracy of individual identification across visits. It should be noted that this upper cutoff frequency is greater than what has been suggested for resting state fMRI analyses. In the literature, using band-pass filters with the lower cutoff frequency of 0.005–0.008 Hz and the upper cutoff frequency of 0.08–0.1 Hz has been recommended (Biswal et al., 1995; Obrig et al., 2000; Cordes et al., 2001; Hampson et al., 2002) although there are studies showing higher frequencies may correspond to neuronal activity related to rsFC (Caballero-Gaudes and Reynolds, 2017). The results of our study showed that removing frequency fluctuations >0.1 Hz adversely affected the reliability of functional interactivity features in identifying individuals. In contrast, removing the global signal had a positive impact on the reliability of the analysis methods except for the DTW distance. This finding implies that the global signal may carry information that is detrimental to representing subject-level information, whereas short time scale fluctuations between 5 and 10 s may beneficial for improving subject-level information. Furthermore, these observations suggest that the relative importance of frequency components in rsFI analyses may vary according to the analysis being conducted. The noted interaction between preprocessing steps and different analysis methods remains to be investigated using other patient populations or subject groups.

### 5.3. Cyclicity and Similarity

Inherently, cyclicity analysis and the other methods analyze different temporal patterns. Cyclicity analysis gauges the temporal ordering of time series, capturing the cyclic interactions amongst time series whereas zero-lag correlation, lagged correlation, and DTW distance measure the similarity between time series. The fMRI time series has serial dependency due to the slow nature of hemodynamic response (Christova et al., 2011; Bright et al., 2017). Although preprocessing procedures, such as de-trending and filtering whiten the time series to some extent and reduce their auto-correlation (Arbabshirani et al., 2014), the serial correlation still remains an issue, suggesting spurious results in correlation-based analyses. In addition, zero-lag correlation does not consider phase shift between time series, and thereby ignores the interactivity information manifested in lag structure.

As mentioned in section 1, lagged correlation has been used to circumvent the phase problem in zero-lag correlation (Mitra et al., 2014, 2015). However, due to the low temporal resolution of fMRI data, the need for applying interpolation techniques, e.g., parabolic (Mitra et al., 2014), bi-cubic (Shah et al., 2018), complicates the estimation of the optimal time delay and its corresponding correlation. In previous studies (Mitra et al., 2014, 2015), it was assumed that lagged correlation curves exhibit a single peak. Much as this assumption simplifies the estimation of the optimal time delay, the extent of its validity depends on preprocessing steps. Particularly, removing the DC component from time-series (e.g., by band-pass filtering) can introduce multiple peaks in the lagged correlation curves. On the other hand, if low-pass filtering is used as in Mitra et al. (2015), the single-peak observation holds. This is mostly because the resting state fMRI data has small fluctuations and keeping the DC component will dominate the lagged correlation results, leading to single peak curves. Our results showed that zero-lag correlation was slightly more reliable in identifying individual across visits than lagged correlation. The DTW distance, on the other hand, does not assume serial independency in time series, and further takes the phase information into consideration (Meszlényi et al., 2017b). Nevertheless, our results showed that DTW distance was the least reliable measure in identifying individuals.

Cyclicity analysis uses information embedded in lag structure beyond only considering the phase differences between time series. That is, it approximately reconstructs a global temporal ordering of the signals in a multidimensional time series, by capturing and analyzing all pairs of cyclic interactions among its constituent elements. Cyclicity analysis does not assume serial independency in time series and yields features that are invariant to re-parameterizations and translations of signals. This feature may be important in analyzing biophysical signals. Consider the cardiac rhythm: while over a shorter time span one may attribute a “period,” over longer time spans it may vary said period—for example resting heart rate vs. active heart rate. Such variations can be considered as re-parameterizations of the time axis and cyclicity analysis produces features that are invariant under such transformations. Similar to correlation-based analyses, cyclicity analysis was shown to be stable and reliable in identifying individuals across visits.

Each feature examined here has several applications and may perform at some applications better than others. This study reiterates that care must be taken in designing preprocessing pipelines for time series because it highly affects the information embedded in the features. This might be relevant for the studies that aim to use pre-cleaned and/or de-noised versions of BOLD time series from various sources.

### 5.4. Future Work

The cyclicity analysis investigated in this study used pairwaise interactions, but the general machinery of the “signature method” can employ triplets and quadruplets and so on, in the context of path signature. Much as this can investigate intricate relationship between time series, the number of features exponentially grows as the order of the interaction increases. Future work will investigate taming this imposed computational complexity, which will allow for generating and studying features that assess the interactivity in a larger scale than pairs.

The coherency of the spontaneous neural fluctuations changes within time scales of seconds or minutes, denoting an underlying non-stationary process (Chang and Glover, 2010; Christova et al., 2011; Hutchison et al., 2013b; Allen et al., 2014). We are planning to adapt various methods, such as sliding window (Handwerker et al., 2012; Hutchison et al., 2013a; Allen et al., 2014) or dynamic mode decomposition (Brunton et al., 2016) to explore dynamic rsFI. Moreover, we plan to take advantage of both static and dynamic analyses to further evaluate and investigate the enhancement of the group-level information within the rsFI features to facilitate identifying subject groups. Additionally, to improve the group signatures in rsFI fMRI data, we will evaluate the techniques similar to ones assessed by Amico and Goñi (2018) in which they identified group-level, subject-level, and noise-level information, and improved the individual fingerprint in functional connectomes.

Finally, due to the fact that different analysis techniques capture different aspects of rsFI, future efforts will be targeted toward developing methods that combine the merits of different analyses. Furthermore, as suggested by Finn et al. (2017), we will leverage the techniques and results of the current study to investigate whether other brain states, rather than resting state (e.g., passively watching video or listening to music), can better manifest group-level information.

## Data Availability Statement

The datasets generated for this study are available on request to the corresponding author.

## Ethics Statement

The studies involving human participants were reviewed and approved by Office of the Protection of Human Subjects, University of Illinois at Urbana-Champaign. The patients/participants provided their written informed consent to participate in this study.

## Author Contributions

FH obtained the funding and supervised this study. SS and BZ analyzed the resting state fMRI data. SS and IA conceptualized the study, conducted all data analyses, and wrote the manuscript under the supervision of FH and YB. BZ assisted in interpreting the results and writing the manuscript. YB supervised the research and advised on the mathematical and theoretical framework of the study. All authors contributed to manuscript revision, read, and approved the submitted version.

## Conflict of Interest

The authors declare that the research was conducted in the absence of any commercial or financial relationships that could be construed as a potential conflict of interest.
