# Supplementary material for: Comparing Cyclicity Analysis With Pre-established Functional Connectivity Methods to Identify Individuals and Subject Groups Using Resting State fMRI
Source: Front Comput Neurosci. 2020 Jan 20;13:94. doi: 10.3389/fncom.2019.00094 (PMC6984040; doi:10.3389/fncom.2019.00094)
Supplement: Supplementary file 1 [file Data_Sheet_1.pdf]

Supplementary Material

This file includes the tables, figures and results that supplement the presentation in the manuscript. In particular, they provide additional information for the interested reader that is not required for interpreting the conclusions of the main article.

1 SUPPLEMENTARY TABLES AND FIGURES

1.1 Tables

Tables S1 and S2 present information regarding the stability analysis of the features in identifying individuals where the resting state fMRI time series were normalized using their Euclidean norms. These results were almost the same as when the time series were normalized over their standard deviation. Table S1 demonstrates the reliability of the features for different filtering conditions while the global signal was not removed from the time series.

Table S1. The accuracy rate of 1-nearest neighbour classifier to identify individuals across two scan sessions held one week apart with different filter specifications and no GSR. The time series were normalized using their Euclidean norms.

	No GSR		
Feature	No filter	BPF ($0.008 \le f_{pass} \le 0.08$ Hz)	BPF ($0.008 \le f_{pass} \le 0.2$ Hz)
LM	0.63	0.43	0.66
CM	0.53	0.53	0.69
LCM	0.48	0.45	0.64
DM	0.25	0.28	0.27

LM: Lead Matrices; CM: Zero-lag Correlation Matrices; LCM: Lagged Correlation Matrices; DM: Dynamic Time Warping Matrices; BPF: Band-Pass Filter; GSR: Global Signal Regression.

Likewise, Table S2 shows the reliability of the features for different filtering conditions, but the global signal was regressed.

Table S2. The accuracy rate of 1-nearest neighbor classifier to identify individuals across two scan sessions held one week apart with different filter specifications and GSR. The time series were normalized using their norm.

	With GSR		
Feature	No filter	BPF ($0.008 \le f_{pass} \le 0.08$ Hz)	BPF ($0.008 \le f_{pass} \le 0.2$ Hz)
LM	0.80	0.54	0.80
CM	0.78	0.82	0.92
LCM	0.76	0.80	0.90
DM	0.25	0.17	0.17

LM: Lead Matrices; CM: Zero-lag Correlation Matrices; LCM: Lagged Correlation Matrices; DM: Dynamic Time Warping Matrices; BPF: Band-Pass Filter; GSR: Global Signal Regression.

1.2 Figures

This section provides visualization of the deep learning architectures used in this paper.

1.2.1 Deep Learning—CNN

For the convolutional neural network, the input was a 33×33 matrix, followed by two convolutional layers. The first convolutional layer had 33 row-wise filter of 1×33, leading to a 3-D output of $33 \times 1 \times 33$. The second convolutional layer had 33 column-wise filter of 33×1, leading to a 3-D output of $1 \times 1 \times 33$. Since shift invariance is not a crucial factor for our application, no pooling layers were used. The second convolutional layer was followed by a fully-connected layer with 33 hidden neurons. A softmax unit was used to estimate the probability distribution of the tinnitus and healthy control groups. Rectified linear units (ReLU) in both convolutional and fully-connected layers were used to introduce non-linearity to the model. A stride of 1 and padding of "valid" were set for both convolutional layers. Cross-entropy was used as the loss function and the Adam optimizer was employed to minimize the loss function over the training data.

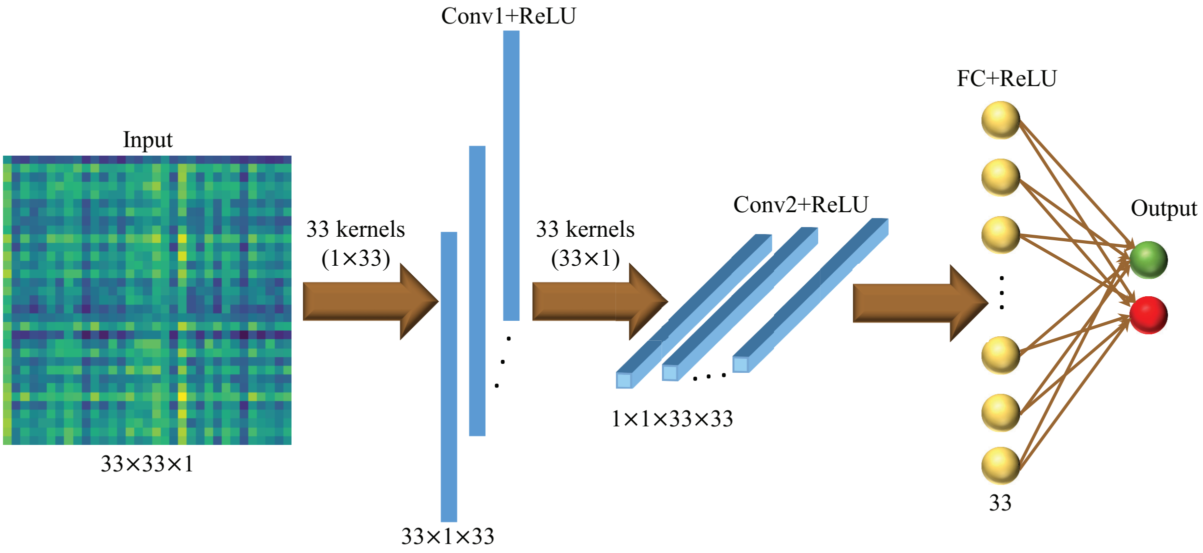

Figure S1: The different layers in the convolutional neural network, a binary classifier; Conv and FC stand for the convolutional layer and fully-connected layer, respectively.

1.2.2 Deep Learning—VAE

An auto-encoder is a neural network consisting of two parts. The first part is an encoder, which is trained to learn a "sparser" or more "efficient" representation of the input data in some latent space. The second part is a decoder, which is given some sampling from the latent space, re-constructs the input fed to the encoder. When a probability distribution is imposed on the structure of the latent space, the resulting auto-encoder is a called a variational encoder. Figure S2 visualizes the major parts of the VAE architecture used in this paper.

1.3 Nonlinear dimensionality reduction

This section shows further examples of the results of non-linear dimensionality reduction from Section 4.2.2 of the manuscript. Using t-SNE, the same trend was observed for LCM as for CM and DM, i.e., there was a separation into two clusters of naturally high correlation values and high anti-correlation values. However this trend was diminished on the application of GSR for LCM. The lead matrices, exhibited no such clustering patterns. A similar trend held under nonlinear dimensionality reduction using the ISOMAP

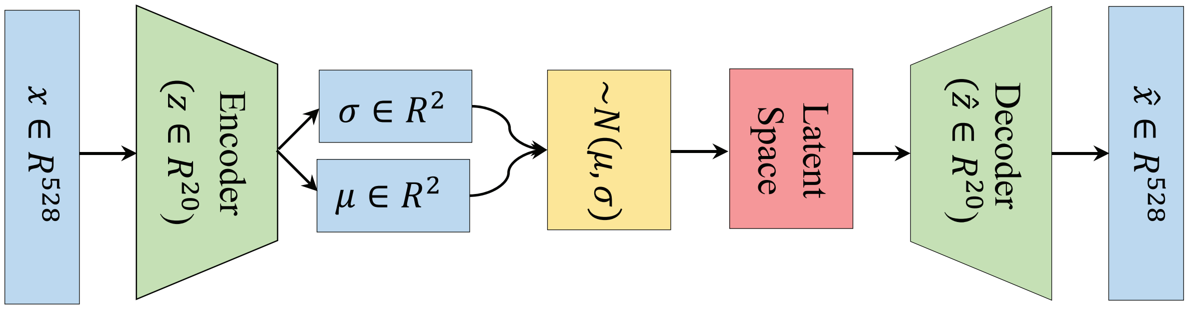

Figure S2: Conceptual visualization of the various steps in the variational auto-encoder.

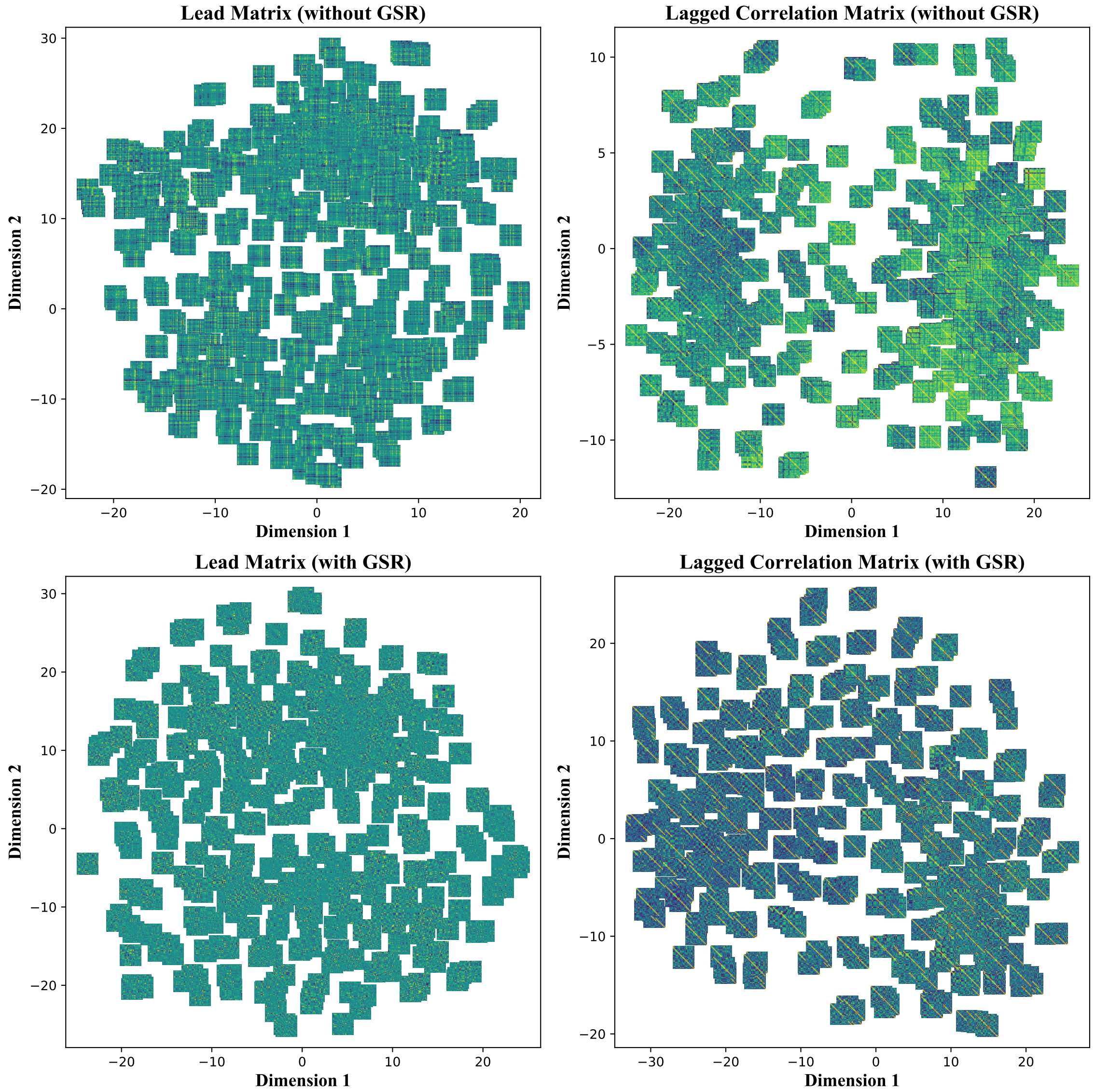

Figure S3: T-distributed Stochastic Neighbor Embedding (t-SNE)—Nonlinear dimensionality reduction to two dimensions of the lead and lagged correlation matrices without and with global signal regression (GSR). The resting state fMRI data were band-pass filtered ($0.008 \leq f_{pass} \leq 0.2$) before extracting the features. Each observation is depicted with its corresponding feature matrix.

algorithm. As noted in the main text, now instead of clustering into groups, the features were peppered along a continuum with the highly correlated and anti-correlated features at the extremals.

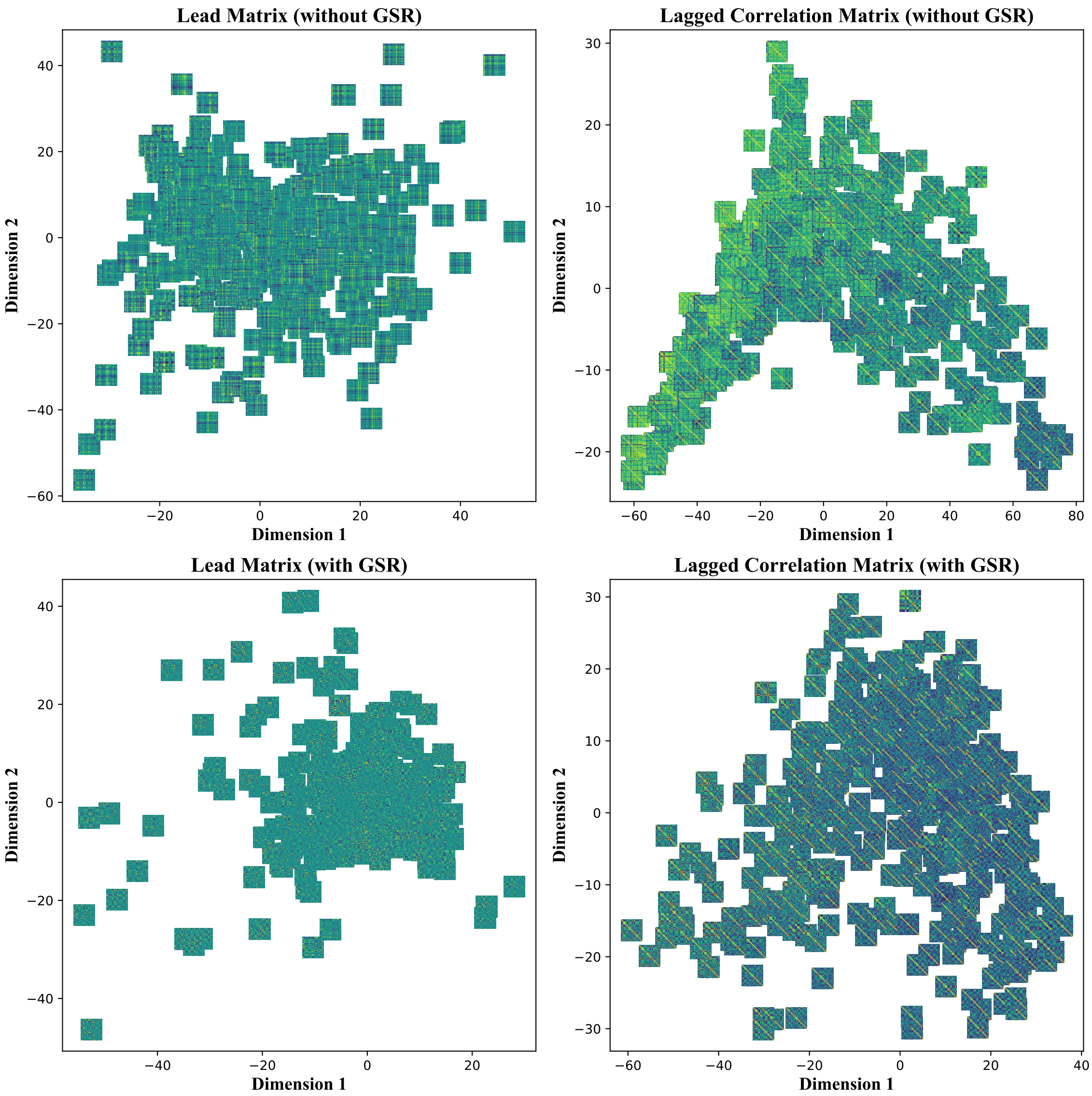

Figure S4: Isomap—Nonlinear dimensionality reduction to two dimensions of the lead and lagged correlation matrices without and with global signal regression (GSR). The resting state fMRI data were band-pass filtered ($0.008 \leq f_{pass} \leq 0.2$) before extracting the features. Each observation is depicted with its corresponding feature matrix.
